# Cu-catalyzed late-stage diversification and anti-proliferative activity evaluation of evodiamine

**DOI:** 10.1039/d5ra09118g

**Published:** 2026-03-10

**Authors:** Jing Wang, Yang Yang, Ming-Li Zhou, Feng Gao, Jin-Bu Xu, Lian Sun

**Affiliations:** a Sichuan Engineering Research Center for Biomimetic Synthesis of Natural Drugs, School of Life Science and Engineering, Southwest Jiaotong University Chengdu 610031 People's Republic of China gaof@swjtu.edu.cn xujinbu@swjtu.edu.cn liansun@swjtu.edu.cn

## Abstract

A series of *N*-13-arylated evodiamine derivatives (3a–3v) were designed and synthesized *via* a Cu-catalyzed late-stage diversification of the bioactive natural product evodiamine. The antitumor activities of these synthesized derivatives against HCT-116, 4T1, and SU-DHL-6 cells were evaluated *in vitro*, demonstrating that direct introduction of aryl groups at the *N*-13 position through C–*N* coupling led to unsatisfactory cytotoxicity. Based on previous studies, the preliminary structure–activity relationship analysis indicated that inserting a suitable pharmacophore fragment between the *N*-13 position and the introduced aryl group can enhance the anti-tumor activity of *N*-13 evodiamine derivatives containing aromatic functional groups. This study provides helpful insights for the further development of antitumor evodiamine analogues.

## Abstract 2

For *N*13 evodiamine analogues containing aromatic groups, inserting a suitable pharmacophore fragment between *N*13 position and introduced aryl group is beneficial for anti-tumor activity.

## Graphical abstract

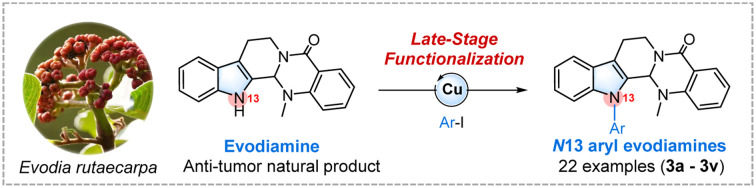

## Introduction

**Fig. 1:**
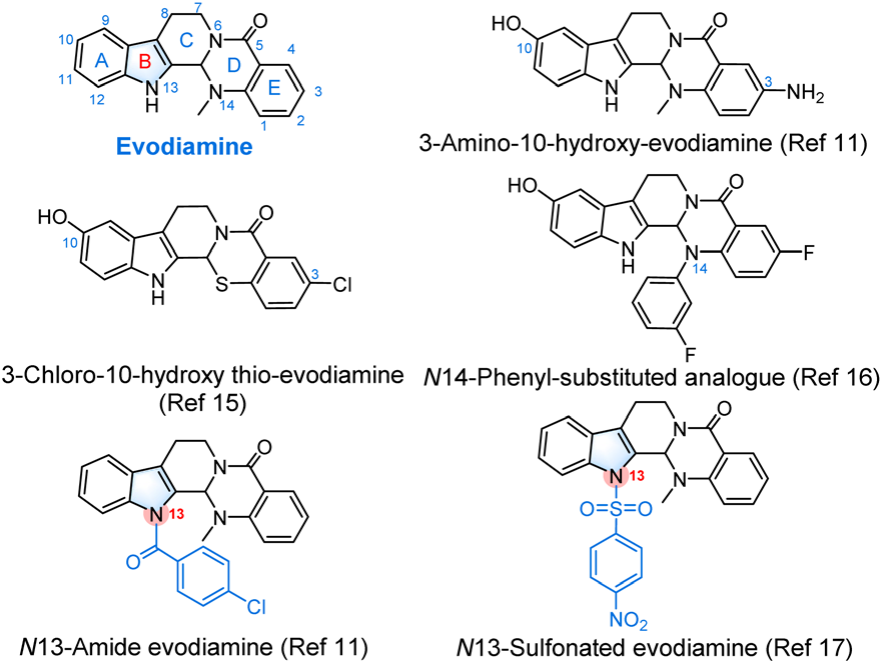
Structure of evodiamine and its bioactive derivatives.

Natural products are considered an invaluable source for new drug discovery.^1,2^ Evodiamine (1), an indoloquinazoline alkaloid isolated from the fruit of the traditional Chinese medicinal plant *Evodia rutaecarpa* (Rutaceae), exhibits a broad range of pharmacological properties, including analgesic,^3,4^ anti-inflammation,^5^ antibacterial^6^ and anti-Alzheimer's disease activities.^7^ Among the diverse pharmacological effects, its multi-targeting antitumor effect has recently received considerable attention in the field of medicinal chemistry.^8,9^ It has been demonstrated that Top I and Top II are the common targets for the antitumor activity of evodiamine and its analogues.^10–13^ Unfortunately, the limited antitumor potency and poor water solubility significantly hamper the clinical development of evodiamine. In light of this, researchers have conducted the structural optimization of evodiamine with the purpose of exploring novel bioactive molecules in recent years.^14^ A series of multi-targeted evodiamine analogues were found (such as 3-amino-10-hydroxy-evodiamine,^11^ 3-chloro-10-hydroxy-thio-evodiamine,^15^ and *N*14-phenyl-substituted evodiamine;^16^Fig. 1), which had improved antitumor potency, Top I or Top II inhibitory activity, and metabolic stability. Notably, *N*-13-amide and sulfonated derivatives also present significantly enhanced anticancer activity,^11,17^ indicating that the indole-NH is a good modifiable active site.

**Fig. 2:**
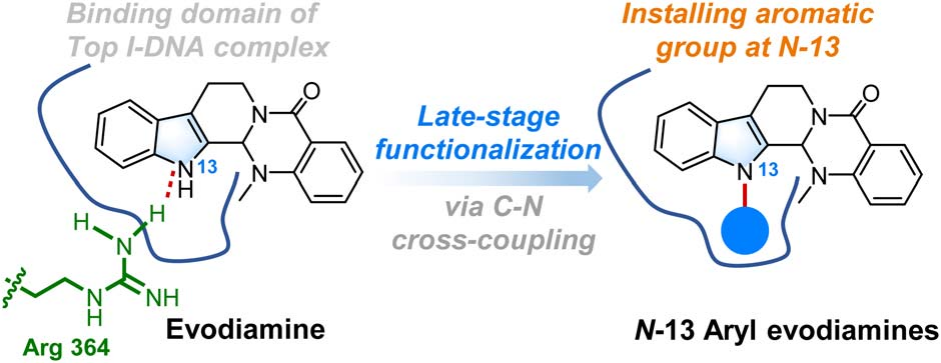
Late-stage functionalization of evodiamine in the present work.

Late-stage functionalization has emerged as an effective strategy to rapidly construct diverse molecular libraries, facilitating the discovery of drug-like molecules.^18^ From the perspective of molecular binding models, the A/B/C ring system in evodiamine intercalates into the DNA base pairs, enabling the molecule with the “L type” conformation to bind to the Top I–DNA complex.^10^ Although the indole-NH forms a hydrogen bond with Arg-364, the B ring is situated in the major groove of the Top I–DNA complex, suggesting that *N*-13 is a promising site for modification. Therefore, we propose the direct introduction of an aromatic group at the *N*-13 position *via* C–*N* coupling,^19,20^ which might fit into the major groove of the Top I–DNA complex and increase the binding affinity by generating π–π bonding interactions (Fig. 2). As part of our continuing efforts to develop potential antitumor bioactive molecules,^21–23^ the present work reports the Cu-catalyzed late-stage diversification of the natural drug evodiamine to efficiently generate an *N*-13 derivative chemical library. A series of new *N*-13-aryl evodiamine derivatives were synthesized *via* a Cu-catalyzed Ullmann C–*N* cross-coupling reaction. The cytotoxicity of the synthesized derivatives against HCT116, 4T1, and SU-DHL-6 cells was further evaluated. This work would contribute to the understanding of the anti-tumor structure–activity relationship of evodiamine.

## Results and discussion

### Chemistry

At the initial stage of the investigation, we attempted to introduce an aromatic group into the *N*-13 position of evodiamine through a Buchwald–Hartwig coupling reaction. However, after screening various Pd-catalyzed reaction systems,^24^ the desired C–*N* coupling failed to occur between evodiamine and aromatic bromide, which might be affected by steric hindrance. Therefore, the present work commenced with the preparation of *N*-13-arylated analogues (3a–3v) *via* a Cu-catalyzed Ullmann-type C–*N* cross-coupling reaction of evodiamine (1) with aryl iodides (2a–2v).^25–27^ Evodiamine (1) and iodobenzene (2a) were employed as model substrates to explore the optimal coupling reaction conditions. With KOH as the base, different Cu catalysts were tested. As shown in Table 1, without the aid of a ligand, treatment of substrates 1 and 2a with various Cu catalysts and KOH as the base in DMSO for 20 h afforded the corresponding product 3a bearing the coupled C–*N* bond at the *N*-13 position (entries 1–8). The preliminary screening showed that CuBr was the best catalyst (entry 2), providing product 3a in 41% yield. To our delight, the yield of 3a was further enhanced to 60% when *N*,*N*′-dimethyl-1,2-ethanediamine (DMEDA) was added as the ligand (entry 9). Subsequently, other bases were also investigated. A maximum yield of 81% was obtained using NaOMe as the base (entry 10). Hence, the best conditions are shown in entry 10 for this Ullmann-type C–*N* coupling reaction.

**Table 1:** Reaction condition optimization^a^

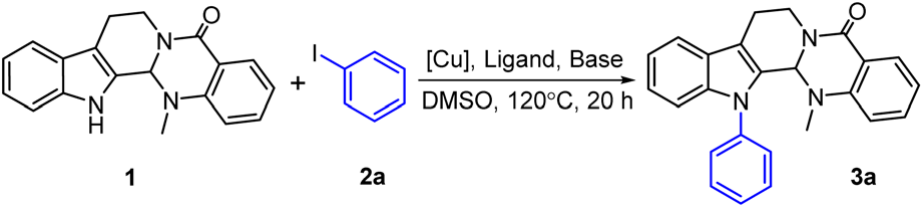				
Entry	Catalyst	Ligand	Base	Yield^b^ (%)
1	Cu_2_O	—	KOH	35
2	CuBr	—	KOH	41
3	Cu(OAc)_2_	—	KOH	32
4	Cu	—	KOH	30
5	CuCl	—	KOH	10
6	CuO	—	KOH	12
7	CuCN	—	KOH	35
8	CuCl_2_	—	KOH	10
9	CuBr	DMEDA	KOH	60
10	CuBr	DMEDA	NaOMe	81

a Conditions: the reaction was conducted with 1 (0.1 mmol, 1 equiv.) and 2a (0.2 mmol, 2 equiv.) in 1 mL DMSO.

b Isolated yield.

**Scheme 1:**
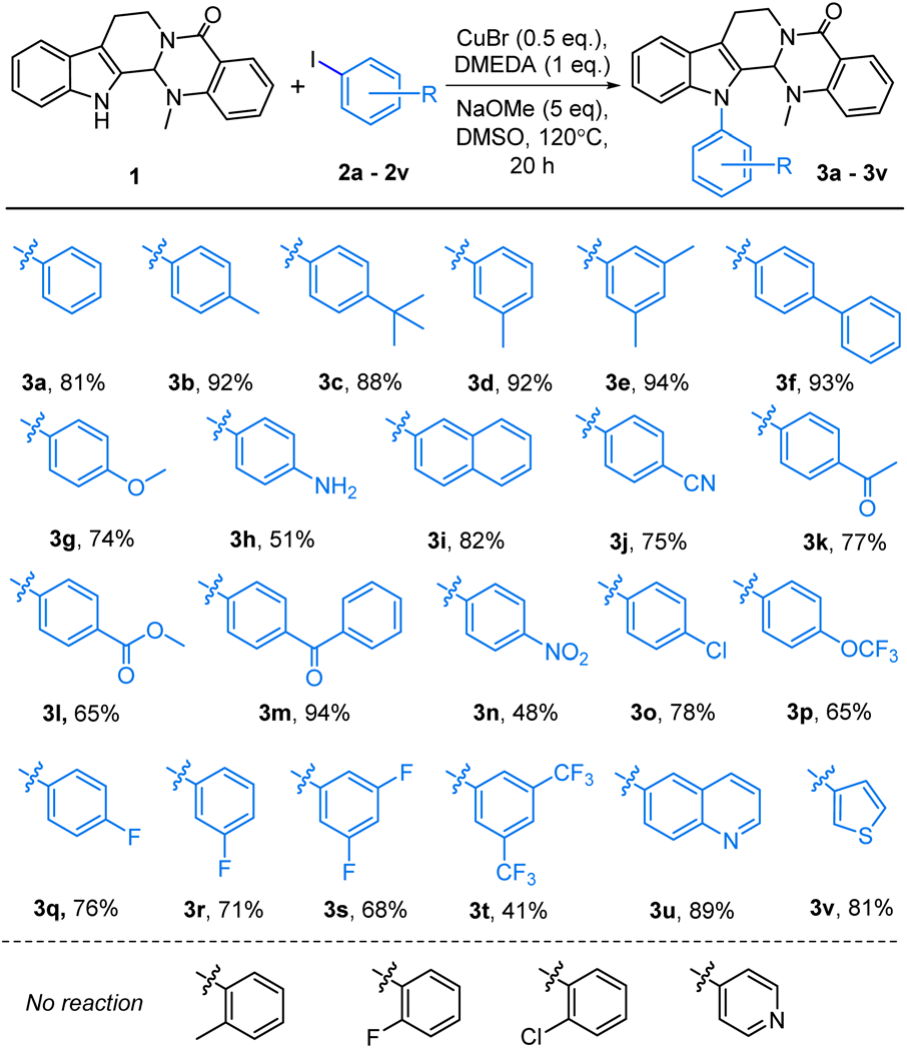
Cu-catalyzed synthesis of *N*-13-aryl evodiamine derivatives.

With the optimized reaction conditions, the substrate scope was explored. A variety of aryl iodides provided the corresponding products in good to excellent yields (Scheme 1). The substrates 2b–2h with electron-donating groups (including methyl, *tert*-butyl, phenyl, methoxyl, and amino) on the benzene ring participated in the coupling reaction to generate the target compounds 3b–3h in yields of 51–94%. Benzene rings bearing electron-withdrawing groups (such as cyano, acyl, acyloxy, nitro, and halogen) gave the corresponding products 3j–3t in 41–94% yields. A variety of functional groups at *para*- or *meta*-substituted positions showed no significant impact on the yields of the target products. However, when using substrates containing *ortho*-position substituents (such as 2-iodotoluene, 2-fluoroiodobenzene, and 2-chloro-iodobenzene) under the same reaction conditions, the coupling products were not generated, which could be explained by the steric hindrance of the aryl iodides. 2-Iodonaphthalene (2i) and 6-iodoquinoline (2u) provided aromatic products 3i and 3u in good yields of 82% and 89%, respectively. Furthermore, sulfur- and nitrogen-containing heteroaryl iodides were also investigated, revealing that 3-iodothiophene (2v) smoothly participated in the reaction to give compound 3v in 81% yield. However, the reaction of 1 with 4-iodopyridine was unsuccessful. This phenomenon might be due to the low electron cloud density of the *para*-position of the pyridine-nitrogen, which reduces the reactivity of the substrate.

### Bioactivity

All the synthesized derivatives (3a–3v) were evaluated for their cytotoxic activity against HCT-116 (human colorectal adenocarcinoma cells), 4T1 (murine breast cancer cells), and SU-DHL-6 (human lymphoma cells). The antiproliferative activities of these compounds are summarized in Table 2. Unfortunately, only a few the synthesized derivatives showed moderate antiproliferation activity against HCT-116, 4T1, and SU-DHL-6 cells at 12.5 µM or 25 µM. For compounds 3b–3h possessing electron-withdrawing groups, only compound 3d exhibited slightly increased potency against 4T1 cells (inhibition ratio of 42.7% at 25 µM) compared with evodiamine (inhibition ratio of 35.5% at 25 µM). In contrast, some compounds possessing electron-withdrawing groups on the benzene ring had higher cytotoxicity than those containing electron-donating groups. In particular, introducing halogen atoms contributed to maintaining or enhancing cytotoxicity. For example, 3n had similar activity to the precursor against the SU-DHL-6 cell line with an inhibition ratio of 83.5% at 12.5 µM. Compounds 3o, 3p, 3r, and 3t showed enhanced cytotoxicity against the 4T1 cell line (inhibition ratios of 42.3%, 57.0%, 53.8%, and 46.5%, respectively, at 25 µM). Furthermore, the trifluoromethyl derivative 3t exhibited good activity against HCT116 and SU-DHL-6 cells. It was speculated that the trifluoromethyl group might have beneficial effects on the target protein *via* weak hydrogen bonding or halogen bonding.

**Table 2:** Antiproliferative activity of compounds 3a–3v

Compound	Inhibition rate^a^ (%)		
	HCT116^b^	4T1^b^	SU-DHL-6^c^
3a	7.7 ± 0.2	19.6 ± 3.4	37.4 ± 4.7
3b	0.3 ± 0.9	17.1 ± 6.6	56.9 ± 8.1
3c	16.1 ± 5.6	37.1 ± 4.4	74.6 ± 3.0
3d	7.3 ± 4.7	42.7 ± 5.1	71.1 ± 5.1
3e	13.0 ± 1.8	26.3 ± 5.4	56.0 ± 3.7
3f	12.1 ± 0.8	12.5 ± 0.6	66.4 ± 2.2
3g	2.4 ± 5.5	37.1 ± 1.7	37.5 ± 3.9
3h	7.1 ± 4.6	9.2 ± 1.8	45.5 ± 5.2
3i	3.4 ± 3.7	6.6 ± 2.7	53.5 ± 8.0
3j	30.4 ± 4.3	65.7 ± 2.3	71.8 ± 4.7
3k	25.2 ± 4.3	46.6 ± 4.1	54.7 ± 3.4
3l	7.2 ± 4.6	6.5 ± 2.6	58.6 ± 3.3
3m	0.1 ± 0.1	10.2 ± 7.2	59.3 ± 3.2
3n	28.2 ± 1.0	38.9 ± 2.2	83.5 ± 2.7
3o	17.7 ± 2.8	42.3 ± 3.4	70.8 ± 1.9
3p	32.0 ± 0.6	57.0 ± 6.2	59.3 ± 3.9
3q	2.5 ± 2.5	21.3 ± 3.7	34.0 ± 6.9
3r	15.0 ± 0.9	53.8 ± 4.7	38.3 ± 4.3
3s	45.6 ± 1.4	11.0 ± 0.9	63.5 ± 2.7
3t	69.9 ± 0.8	46.5 ± 1.4	88.2 ± 2.7
3u	29.6 ± 0.7	21.0 ± 7.0	72.9 ± 3.9
3v	0.1 ± 0.6	48.9 ± 2.2	48.4 ± 8.7
Evo	40.4 ± 1.6	35.5 ± 0.9	85.0 ± 1.3

a Data are expressed as means ± SD (*n* = 3).

b 25 µM.

c 12.5 µM.

**Fig. 3:**
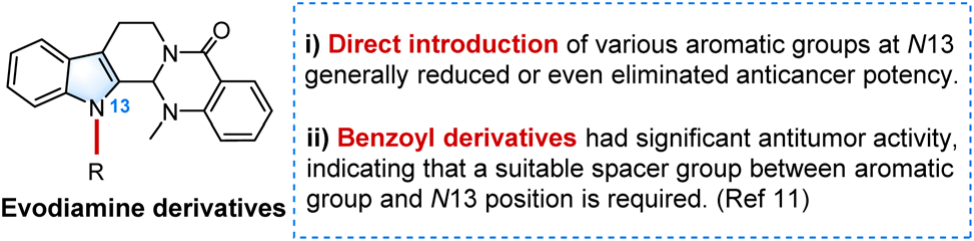
SARs of *N*-13 evodiamine derivatives for cytotoxicity.

Overall, the direct attachment of aromatic groups to the *N*-13 position of evodiamine generally reduced or even eliminated its anticancer potency. Although the introduction of an aromatic group may enhance base-stacking interactions with Top I, it concurrently interferes with the crucial hydrogen bond between the indole-NH and Arg-364, which is likely a major reason for the diminished activity. In contrast, previously reported *N*-13-amide and *N*-13-sulfonamide evodiamine derivatives exhibited significant antitumor effects.^11,17^ Thus, the incorporation of a suitable spacer group (*e.g.*, an acyl group) between the *N*-13 position and the aromatic ring is necessary to preserve the essential hydrogen bond with Top I and thereby enhance the potency of the *N*-13-modified evodiamine derivatives (Fig. 3).

## Conclusions

In conclusion, the late-stage functionalization of the bioactive natural product evodiamine (1) was achieved. Twenty-two *N*-13-aryl evodiamine derivatives (3a–3v) were synthesized in good to excellent yields *via* a Cu-catalyzed Ullmann-type C–*N* cross-coupling reaction between 1 and aryl iodides. Although most compounds showed low cytotoxicity, a preliminary structure–activity relationship was established based on previous reports. It was found that direct arylation at the *N*-13 position reduced the antitumor activity, whereas incorporating a suitable pharmacophore spacer between the *N*-13 atom and the aryl ring proved to be a beneficial strategy for the structural optimization of evodiamine analogues. This study enhances the understanding of the antitumor structure–activity relationship of evodiamine and provides helpful insights for its future structural optimization.

## Experimental

### General

Evodiamine with a purity of >98% was purchased from Shanghai McLean Biochemical Technology Co., Ltd. The reagents were purchased from commercial sources and used directly without purification. Reactions were monitored by TLC on silica gel GF_245_ plates, and the spots were visualized by UV irradiation (254 nm). Compounds were purified by flash column chromatography equipped with silica gel (200–300 mesh). ^1^H and ^13^C NMR spectra were recorded in CDCl_3_ or CD_3_OD on a Bruker AV 400 or 600 nuclear magnetic resonance instrument. (+)-HRESIMS spectra were obtained using a Waters ACQUITY UPLC/Xevo G2-S Q-Tof mass spectrometer with an electro-spray ionization (ESI) ionization mode, equipped with a Waters ACQUITYUPLC system.

### General procedure for the synthesis of *N*-13-aryl-evodiamine derivatives (3a–3v)

A solution of evodiamine (1, 0.1 mmol, and 1 equiv.), aryl iodide (2a–2v, 0.2 mmol, and 2 equiv.), CuBr (0.05 mmol and 0.5 equiv.), NaOMe (0.5 mmol and 5 equiv.), DMEDA (0.1 mmol and 1 equiv.), and DMSO (1 mL) was heated to 120 °C under an argon atmosphere. After 20 h, the reaction was quenched with H_2_O, extracted with EtOAc, dried over Na_2_SO_4_, filtered, and concentrated under reduced pressure. The crude product was purified by column chromatography to afford the desired products 3a–3v.

Compound 3a, yellow amorphous powder, 81% yield; ^1^H NMR (600 MHz, CDCl_3_) *δ*: 8.05 (dd, *J* = 7.8, 1.2 Hz, 1H), 7.67–7.66 (m, 1H), 7.51–7.42 (m, 5H), 7.35–7.33 (m, 1H), 7.25–7.23 (m, 3H), 7.10 (td, *J* = 7.2, 1.2 Hz, 1H), 6.74 (dd, *J* = 7.8, 1.2 Hz, 1H), 5.88 (s, 1H), 4.95 (ddd, *J* = 12.6, 4.8, 1.8 Hz, 1H), 3.29 (td, *J* = 11.4, 4.2 Hz, 1H), 3.11–3.08 (m, 1H), 3.03–2.97 (m, 1H), and 2.37 (s, 3H); ^13^C NMR (100 MHz, CDCl_3_) *δ*: 164.8, 150.7, 138.8, 137.6, 132.9, 129.51, 129.49, 128.8, 128.0, 127.3, 126.0, 123.6, 123.5 × 2, 122.2, 120.6, 119.0, 115.2, 110.9, 68.0, 39.5, 36.4, and 20.7; HRESIMS (*m*/*z*): 380.1776 [M + H]^+^ (calcd for C_25_H_22_N_3_O, 380.1763).

Compound 3b, white amorphous powder, 92% yield; ^1^H NMR (600 MHz, CDCl_3_) *δ*: 8.06 (dd, *J* = 7.8, 1.6 Hz, 1H), 7.67–7.65 (m, 1H), 7.37–7.27 (m, 5H), 7.24–7.21 (m, 3H), 7.11 (td, *J* = 7.8, 1.2 Hz, 1H), 6.80 (d, *J* = 8.4 Hz, 1H), 5.85 (s, 1H), 4.96 (ddd, *J* = 12.6, 4.8, 1.8 Hz, 1H), 3.28 (td, *J* = 11.4, 4.2 Hz, 1H), 3.11 (dd, *J* = 15.0, 3.6 Hz, 1H), 3.02–2.96 (m, 1H), 2.44 (s, 3H), and 2.40 (s, 3H); ^13^C NMR (100 MHz, CDCl_3_) *δ*: 164.8, 150.7, 139.0, 137.9, 134.9, 132.8, 130.1, 129.6, 128.8, 127.2, 125.9, 123.5, 123.31, 123.27, 122.0, 120.4, 118.9, 115.0, 111.0, 67.9, 39.5, 36.3, 21.3, and 20.7; HRESIMS (*m*/*z*): 394.1923 [M + H]^+^ (calcd for C_26_H_24_N_3_O, 394.1919).

Compound 3c, white amorphous powder, 88% yield; ^1^H NMR (600 MHz, CDCl_3_) *δ*: 8.06 (dd, *J* = 7.8, 1.6 Hz, 1H), 7.66 (dd, *J* = 6.6, 1.8 Hz, 1H), 7.49–7.34 (m, 5H), 7.28–7.20 (m, 3H), 7.11 (td, *J* = 8.4, 1.2 Hz, 1H), 6.73 (d, *J* = 8.4 Hz, 1H), 5.87 (s, 1H), 4.95 (ddd, *J* = 12.6, 4.8, 1.8 Hz, 1H), 3.29 (td, *J* = 12.0, 4.2 Hz, 1H), 3.11–3.08 (m, 1H), 3.02–2.96 (m, 1H), 2.37 (s, 3H), and 1.38 (s, 9H); ^13^C NMR (100 MHz, CDCl_3_) *δ*: 164.8, 151.1, 150.7, 138.9, 134.9, 132.8, 129.6, 128.8, 126.8, 126.3, 125.9, 123.5, 123.3 × 2, 122.0, 120.4, 118.9, 114.9, 111.1, 68.0, 39.5, 36.3, 34.9, 31.5, and 20.7; HRESIMS (*m*/*z*): 436.2391 [M + H]^+^ (calcd for C_29_H_30_N_3_O, 436.2389).

Compound 3d, yellow amorphous powder, 92% yield; ^1^H NMR (400 MHz, CDCl_3_) *δ*: 8.06 (dd, *J* = 7.8, 1.6 Hz, 1H), 7.68–7.65 (m, 1H), 7.40–7.34 (m, 3H), 7.26–7.21 (m, 5H), 7.13 (td, *J* = 7.6, 1.2 Hz, 1H), 6.79 (d, *J* = 8.0 Hz, 1H), 5.86 (s, 1H), 4.97 (ddd, *J* = 12.6, 4.8, 1.8 Hz, 1H), 3.31 (td, *J* = 12.0, 4.4 Hz, 1H), 3.13–3.07 (m, 1H), 3.05–2.96 (m, 1H), and 2.41 (s, 6H); ^13^C NMR (150 MHz, CDCl_3_) *δ*: 164.8, 150.6, 139.4, 138.9, 137.5, 132.8, 129.6, 129.2, 128.83, 128.81, 128.0, 125.9, 124.4, 123.4, 123.33, 123.26, 121.9, 120.5, 118.9, 115.1, 111.0, 68.1, 39.6, 36.4, 21.5, and 20.7; HRESIMS (*m*/*z*): 394.1934 [M + H]^+^ (calcd for C_26_H_24_N_3_O, 394.1919).

Compound 3e, yellow amorphous powder, 94% yield; ^1^H NMR (600 MHz, CDCl_3_) *δ*: 8.06 (dd, *J* = 7.8, 1.6 Hz, 1H), 7.66–7.65 (m, 1H), 7.39–7.36 (m, 1H), 7.24–7.20 (m, 4H), 7.12–7.05 (m, 3H), 6.81 (d, *J* = 8.2 Hz, 1H), 5.82 (s, 1H), 4.95 (ddd, *J* = 12.6, 4.8, 1.8 Hz, 1H), 3.28 (td, *J* = 12.0, 4.2 Hz, 1H), 3.10–3.07 (m, 1H), 3.03–2.98 (m, 1H), 2.43 (s, 3H), and 2.37 (s, 6H); ^13^C NMR (150 MHz, CDCl_3_) *δ*: 164.8, 150.6, 139.02, 138.98, 137.3, 132.8, 129.7, 128.8, 125.9, 125.2, 123.3, 123.2, 123.1, 121.7, 120.4, 118.9, 115.0, 111.1 × 2, 68.1, 39.7, 36.4, 21.4, and 20.7; HRESIMS (*m*/*z*): 408.2084 [M + H]^+^ (calcd for C_27_H_26_N_3_O, 408.2076).

Compound 3f, yellow amorphous powder, 93% yield; ^1^H NMR (600 MHz, CDCl_3_) *δ*: 8.07 (dd, *J* = 7.8, 1.6 Hz, 1H), 7.72 (d, *J* = 8.4 Hz, 2H), 7.69–7.66 (m, 3H), 7.52–7.48 (m, 4H), 7.41 (t, *J* = 7.2 Hz, 1H), 7.36–7.32 (m, 2H), 7.28 (td, *J* = 7.2, 1.8 Hz, 1H), 7.26–7.23 (m, 1H), 7.13 (t, *J* = 7.2 Hz, 1H), 6.78 (d, *J* = 7.8 Hz, 1H), 5.92 (s, 1H), 4.97 (ddd, *J* = 12.6, 4.8, 1.8 Hz, 1H), 3.31 (td, *J* = 12.0, 4.2 Hz, 1H), 3.13–3.09 (m, 1H), 3.04–2.98 (m, 1H), and 2.41 (s, 3H); ^13^C NMR (150 MHz, CDCl_3_) *δ*: 164.8, 150.7, 140.8, 140.2, 138.9, 136.8, 132.9, 129.6, 129.1, 128.8, 128.1, 127.8, 127.6, 127.2, 126.1, 123.6, 123.5, 123.5, 122.2, 120.7, 119.1, 115.4, 111.0, 68.0, 39.5, 36.4, and 20.7; HRESIMS (*m*/*z*): 456.2079 [M + H]^+^ (calcd for C_31_H_26_N_3_O, 456.2076).

Compound 3g, yellow amorphous powder, 74% yield; ^1^H NMR (600 MHz, CDCl_3_) *δ*: 8.05 (dd, *J* = 7.8, 1.6 Hz, 1H), 7.66–7.65 (m, 1H), 7.37–7.31 (m, 3H), 7.25–7.21 (m, 2H), 7.20–7.17 (m, 1H), 7.11 (td, *J* = 8.4, 1.2 Hz, 1H), 7.00–6.98 (m, 2H), 6.81 (d, *J* = 8.4 Hz, 1H), 5.81 (s, 1H), 4.93 (ddd, *J* = 12.6, 4.8, 1.8 Hz, 1H), 3.87 (s, 3H), 3.27 (td, *J* = 11.4, 4.2 Hz, 1H), 3.10–3.07 (m, 1H), 3.01–2.96 (m, 1H), and 2.40 (s, 3H); ^13^C NMR (100 MHz, CDCl_3_) *δ*: 164.8, 159.3, 150.7, 139.3, 132.9, 130.2, 129.8, 128.8, 128.7, 125.8, 123.5, 123.4, 123.3, 122.1, 120.4, 118.9, 114.8, 114.6, 110.9, 68.0, 55.7, 39.6, 36.4, and 20.7; HRESIMS (*m*/*z*): 410.1862 [M + H]^+^ (calcd for C_26_H_24_N_3_O_2_, 410.1869).

Compound 3h, yellow amorphous powder, 51% yield; ^1^H NMR (600 MHz, CD_3_OD) *δ*: 7.88–7.87 (m, 1H), 7.59 (d, *J* = 7.4 Hz, 1H), 7.38–7.35 (m, 1H), 7.15–7.11 (m, 3H), 7.09 (t, *J* = 6.0 Hz, 2H), 7.04 (t, *J* = 7.2 Hz, 1H), 6.80–6.79 (m, 3H), 5.86 (s, 1H), 4.78 (ddd, *J* = 12.6, 4.8, 1.8 Hz, 1H), 3.26 (td, *J* = 12.0, 4.2 Hz, 1H), 3.06 (dd, *J* = 15.6, 4.2 Hz, 1H), 2.97–2.90 (m, 1H), and 2.36 (s, 3H); ^13^C NMR (150 MHz, CDCl_3_) *δ*: 164.9, 150.7, 146.4, 139.4, 132.8, 130.0, 128.9, 128.7, 128.1, 125.8, 123.4, 123.1 × 2, 121.9, 120.3, 118.9, 115.5, 114.5, 111.0, 68.0, 39.7, 36.3, and 20.7; HRESIMS (*m*/*z*): 395.1872 [M + H]^+^ (calcd for C_25_H_23_N_4_O, 395.1874).

Compound 3i, yellow amorphous powder, 82% yield; ^1^H NMR (600 MHz, CDCl_3_) *δ*: 8.05 (dd, *J* = 7.8, 1.2 Hz, 1H), 8.00–7.93 (m, 3H), 7.87 (s, 1H), 7.73–7.70 (m, 1H), 7.58–7.55 (m, 3H), 7.29–7.25 (m, 4H), 7.08 (t, *J* = 7.5 Hz, 1H), 6.68 (d, *J* = 8.0 Hz, 1H), 5.94 (s, 1H), 5.00–4.96 (ddd, *J* = 12.6, 4.8, 1.8 Hz, 1H), 3.29 (td, *J* = 11.4, 4.2 Hz, 1H), 3.15–3.12 (m, 1H), 3.08–3.02 (m, 1H), and 2.44 (s, 3H); ^13^C NMR (150 MHz, CDCl_3_) *δ*: 164.7, 150.5, 139.0, 135.0, 133.6, 132.8, 132.6, 129.8, 129.3, 128.7, 128.0, 127.9, 126.8, 126.7, 126.0, 125.7, 125.5, 123.5, 123.39, 123.35, 122.0, 120.6, 119.0, 115.4, 110.9, 68.1, 39.5, 36.4, and 20.7; HRESIMS (*m*/*z*): 430.1921 [M + H]^+^ (calcd for C_29_H_24_N_3_O, 430.1919).

Compound 3j, yellow amorphous powder, 75% yield; ^1^H NMR (600 MHz, CDCl_3_) *δ*: 8.06 (dd, *J* = 7.8, 1.6 Hz, 1H), 7.80 (d, *J* = 8.7 Hz, 2H), 7.68–7.67 (m, 1H), 7.61 (d, *J* = 7.9 Hz, 2H), 7.39 (td, *J* = 8.1, 1.6 Hz, 1H), 7.30–7.27 (m, 3H), 7.17 (td, *J* = 7.5, 1.1 Hz, 1H), 6.73 (dd, *J* = 8.1, 1.2 Hz, 1H), 5.89 (s, 1H), 4.96 (ddd, *J* = 12.6, 4.8, 1.8 Hz, 1H), 3.29 (td, *J* = 11.4, 4.2 Hz, 1H), 3.10–3.07 (m, 1H), 3.00–2.94 (m, 1H), and 2.31 (s, 3H); ^13^C NMR (100 MHz, CDCl_3_) *δ*: 164.6, 150.4, 142.0, 138.1, 133.5, 133.2, 128.9 × 2, 127.5, 126.5, 124.3, 124.2, 123.9, 122.7, 121.5, 119.5, 118.4, 116.9, 111.3, 110.6, 67.9, 39.1, 36.4, and 20.6; HRESIMS (*m*/*z*): 405.1719 [M + H]^+^ (calcd for C_26_H_21_N_4_O, 405.1715).

Compound 3k, yellow amorphous powder, 77% yield; ^1^H NMR (600 MHz, CDCl_3_) *δ*: 8.10 (d, *J* = 9.0 Hz, 2H), 8.05 (dd, *J* = 7.8, 1.6 Hz, 1H), 7.68–7.67 (m, 1H), 7.57 (d, *J* = 7.4 Hz, 2H), 7.36–7.33 (m, 1H), 7.30–7.24 (m, 3H), 7.15 (td, *J* = 7.8, 1.2 Hz, 1H), 6.74 (d, *J* = 8.4 Hz, 1H), 5.91 (s, 1H), 4.96–4.92 (ddd, *J* = 12.6, 4.8, 1.8 Hz, 1H), 3.29 (td, *J* = 11.4, 4.2 Hz, 1H), 3.10–3.07 (m, 1H), 3.00–2.95 (m, 1H), 2.67 (s, 3H), and 2.34 (s, 3H); ^13^C NMR (100 MHz, CDCl_3_) *δ*: 197.2, 164.7, 150.5, 142.1, 138.4, 136.1, 133.0, 129.8, 129.1, 128.9, 127.0, 126.4, 124.0, 123.9, 123.8, 122.5, 121.1, 119.3, 116.4, 110.8, 67.9, 39.2, 36.4, 26.8, and 20.7; HRESIMS (*m*/*z*): 422.1872 [M + H]^+^ (calcd for C_27_H_24_N_3_O_2_, 422.1869).

Compound 3l, yellow amorphous powder, 65% yield; ^1^H NMR (600 MHz, CDCl_3_) *δ*: 8.17 (d, *J* = 8.2 Hz, 2H), 8.05 (d, *J* = 7.8 Hz, 1H), 7.67 (d, *J* = 7.6 Hz, 1H), 7.54 (d, *J* = 6.5 Hz, 2H), 7.35 (t, *J* = 7.2 Hz, 1H), 7.28–7.24 (m, 3H), 7.13 (t, *J* = 7.8 Hz, 1H), 6.72 (d, *J* = 8.0 Hz, 1H), 5.91 (s, 1H), 4.96 (dd, *J* = 12.6, 4.8 Hz, 1H), 3.97 (s, 3H), 3.30 (td, *J* = 11.4, 4.2 Hz, 1H), 3.12–3.08 (m, 1H), 3.02–2.96 (m, 1H), and 2.33 (s, 3H); ^13^C NMR (150 MHz, CDCl_3_) *δ*: 166.5, 164.7, 150.5, 142.0, 138.4, 133.0, 131.0, 129.4, 129.2, 128.9, 126.8, 126.4, 123.90, 123.89, 123.7, 122.5, 121.1, 119.3, 116.3, 110.8, 68.0, 52.5, 39.3, 36.4, and 20.7; HRESIMS (*m*/*z*): 438.1815 [M + H]^+^ (calcd for C_27_H_24_N_3_O_3_, 438.1818).

Compound 3m, yellow amorphous powder, 94% yield; ^1^H NMR (600 MHz, CDCl_3_) *δ*: 8.07 (d, *J* = 7.8 Hz, 1H), 7.96 (d, *J* = 7.8 Hz, 2H), 7.85 (dd, *J* = 7.2 Hz, 2H), 7.69 (d, *J* = 7.6 Hz, 1H), 7.66–7.56 (m, 3H), 7.52 (t, *J* = 7.6 Hz, 2H), 7.39–7.36 (m, 2H), 7.34–7.24 (m, 2H), 7.15 (t, *J* = 7.8 Hz, 1H), 6.78 (d, *J* = 8.0 Hz, 1H), 5.95 (s, 1H), 4.95 (ddd, *J* = 12.6, 4.8, 1.8 Hz, 1H), 3.31 (td, *J* = 11.4, 4.2 Hz, 1H), 3.13–3.10 (m, 1H), 3.03–2.97 (m, 1H), and 2.36 (s, 3H); ^13^C NMR (150 MHz, CDCl_3_) *δ*: 195.7, 164.7, 150.6, 141.5, 138.4, 137.4, 136.6, 133.0, 132.8, 131.4, 130.1, 129.2, 128.9, 128.6, 126.7, 126.4, 124.0, 123.9, 123.8, 122.5, 121.1, 119.3, 116.3, 110.9, 68.0, 39.2, 36.4, and 20.6; HRESIMS (*m*/*z*): 484.2042 [M + H]^+^ (calcd for C_32_H_26_N_3_O_2_, 484.2025).

Compound 3n, yellow amorphous powder, 48% yield; ^1^H NMR (400 MHz, CDCl_3_) *δ*: 8.38 (d, *J* = 7.6 Hz, 2H), 8.06 (dd, *J* = 7.8, 1.6 Hz, 1H), 7.69–7.67 (m, 3H), 7.38 (td, *J* = 7.6, 1.6 Hz, 1H), 7.33–7.27 (m, 3H), 7.19 (t, *J* = 7.6 Hz, 1H), 6.74 (d, *J* = 8.0 Hz, 1H), 5.92 (s, 1H), 4.98 (ddd, *J* = 12.6, 4.8, 1.8 Hz, 1H), 3.31 (td, *J* = 11.4, 4.2 Hz, 1H), 3.15–3.05 (m, 1H), 3.04–2.92 (m, 1H), and 2.32 (s, 3H); ^13^C NMR (150 MHz, CDCl_3_) *δ*: 164.6, 150.4, 146.6, 143.7, 138.2, 133.2, 129.0, 127.3, 126.7, 125.1, 124.43, 124.40, 124.0, 122.8, 121.6, 119.6, 117.3, 110.6, 68.0, 39.1, 36.5, and 20.6; HRESIMS (*m*/*z*): 425.1623 [M + H]^+^ (calcd for C_25_H_21_N_4_O_3_, 425.1614).

Compound 3o, yellow amorphous powder, 78% yield; ^1^H NMR (600 MHz, CDCl_3_) *δ*: 8.06 (dd, *J* = 7.8, 1.6 Hz, 1H), 7.68 (d, *J* = 7.2 Hz, 1H), 7.47 (d, *J* = 8.2 Hz, 2H), 7.43–7.30 (m, 3H), 7.26–7.20 (m, 3H), 7.15 (t, *J* = 7.2 Hz, 1H), 6.80 (d, *J* = 7.8 Hz, 1H), 5.83 (s, 1H), 4.94 (ddd, *J* = 12.6, 4.8, 1.8 Hz, 1H), 3.28 (td, *J* = 11.4, 4.2 Hz, 1H), 3.10–3.06 (m, 1H), 3.00–2.95 (m 1H), and 2.37 (s, 3H); ^13^C NMR (100 MHz, CDCl_3_) *δ*: 164.7, 150.6, 138.8, 136.2, 133.8, 133.0, 129.7, 129.4, 128.9, 128.6, 126.1, 123.9, 123.74, 123.71, 122.5, 120.9, 119.2, 115.6, 110.7, 68.0, 39.4, 36.4, and 20.6; HRESIMS (*m*/*z*): 414.1344 [M + H]^+^ (calcd for C_25_H_21_ClN_3_O, 414.1373).

Compound 3p, yellow amorphous powder, 65% yield; ^1^H NMR (600 MHz, CDCl_3_) *δ*: 8.09 (dd, *J* = 7.8, 1.2 Hz, 1H), 7.70–7.69 (m, 1H), 7.74–7.51 (m, 2H), 7.41–7.37 (m, 3H), 7.31–7.26 (m, 3H), 7.18 (t, *J* = 7.2 Hz, 1H), 6.77 (d, *J* = 6.6 Hz, 1H), 5.88 (s, 1H), 4.98 (ddd, *J* = 12.6, 4.8, 1.8 Hz, 1H), 3.32 (td, *J* = 11.4, 4.2 Hz, 1H), 3.15–3.08 (m, 1H), 3.06–2.98 (m, 1H), and 2.37 (s, 3H); ^13^C NMR (100 MHz, CDCl_3_) *δ*: 164.7, 150.6, 148.5 (d, *J* = 2 Hz), 138.7, 136.3, 133.0, 129.4, 128.9, 128.7, 126.1, 124.0, 123.84, 123.76, 122.5, 122.0, 121.9 (d, *J* = 257 Hz), 120.9, 119.2, 115.6, 110.7, 68.1, 39.3, 36.5, and 20.6; HRESIMS (*m*/*z*): 464.1593 [M + H]^+^ (calcd for C_26_H_21_F_3_N_3_O_2_, 464.1586).

Compound 3q, yellow amorphous powder, 76% yield; ^1^H NMR (600 MHz, CDCl_3_) *δ*: 8.05 (dd, *J* = 7.8, 1.6 Hz, 1H), 7.66 (d, *J* = 6.6 Hz, 1H), 7.43–7.35 (m, 3H), 7.26–7.22 (m, 3H), 7.19–7.18 (m, 2H), 7.13 (t, *J* = 7.2 Hz, 1H), 6.78 (d, *J* = 8.0 Hz, 1H), 5.81 (s, 1H), 4.95 (ddd, *J* = 12.6, 4.8, 1.8 Hz, 1H), 3.31 (td, *J* = 11.4, 4.2 Hz, 1H), 3.12–3.09 (m, 1H), 3.02–2.93 (m, 1H), and 2.37 (s, 3H); ^13^C NMR (150 MHz, CDCl_3_) *δ*: 164.8, 162.9 (*J* = 240 Hz), 150.6, 139.0, 133.6 (*J* = 3 Hz), 133.0, 129.6, 129.2, 128.9, 126.0, 123.8, 123.7, 123.6, 122.4, 120.7, 119.1, 116.5 (*J* = 23 Hz), 115.3, 110.7, 68.0, 39.5, 36.4, and 20.6; HRESIMS (*m*/*z*): 398.1661 [M + H]^+^ (calcd for C_25_H_21_FN_3_O, 398.1669).

Compound 3r, yellow amorphous powder, 71% yield; ^1^H NMR (400 MHz, CDCl_3_) *δ*: 8.06 (dd, *J* = 7.8, 1.6 Hz, 1H), 7.67–7.65 (m, 1H), 7.48–7.42 (m, 1H), 7.39 (td, *J* = 8.4, 1.6 Hz, 1H), 7.28–7.22 (m, 5H), 7.17–7.12 (m, 2H), 6.80 (d, *J* = 8.2 Hz, 1H), 5.87 (s, 1H), 4.96–4.92 (ddd, *J* = 12.6, 4.8, 1.8 Hz, 1H), 3.30 (td, *J* = 11.4, 4.2 Hz, 1H), 3.12–3.06 (m, 1H), 3.02–2.94 (m, 1H), and 2.37 (s, 3H); ^13^C NMR (150 MHz, CDCl_3_) *δ*: 164.7, 163.9 (d, *J* = 246 Hz), 150.6, 139.2 (d, *J* = 9 Hz), 138.6, 133.0, 130.7 (d, *J* = 9 Hz), 129.3, 128.9, 126.1, 123.9, 123.79, 123.77, 123.0 (d, *J* = 3 Hz), 122.5, 120.9, 119.2, 115.8, 115.1 (d, *J* = 23 Hz), 114.8 (d, *J* = 23 Hz), 110.8, 68.0, 39.3, 36.4, and 20.6; HRESIMS (*m*/*z*): 398.1676 [M + H]^+^ (calcd for C_25_H_21_FN_3_O, 398.1669).

Compound 3s, yellow amorphous powder, 68% yield; ^1^H NMR (400 MHz, CDCl_3_) *δ*: 8.07 (dd, *J* = 7.8, 1.6 Hz, 1H), 7.66 (d, *J* = 6.8 Hz, 1H), 7.41 (td, *J* = 7.6, 1.6 Hz, 1H), 7.33–7.24 (m, 3H), 7.17 (t, *J* = 7.6 Hz, 1H), 7.08 (d, *J* = 6.4 Hz, 2H), 6.93–6.86 (m, 2H), 5.86 (s, 1H), 4.97 (ddd, *J* = 12.6, 4.8, 1.8 Hz, 1H), 3.28 (td, *J* = 11.4, 4.2 Hz, 1H), 3.11–3.06 (m, 1H), 3.01–2.92 (m, 1H), and 2.37 (s, 3H); ^13^C NMR (150 MHz, CDCl_3_) *δ*: 164.6, 164.1 (d, *J* = 13.5 Hz), 162.5 (d, *J* = 13.5 Hz), 150.6, 140.0 (t, *J* = 12 Hz), 138.4, 133.2, 129.1, 128.9, 126.3, 124.2, 124.1, 124.0, 122.8, 121.3, 119.3, 116.5, 110.8, 103.7 (t, *J* = 24 Hz), 68.0, 39.2, 36.5, and 20.6; HRESIMS (*m*/*z*): 416.1556 [M + H]^+^ (calcd for C_25_H_20_F_2_N_3_O, 416.1574).

Compound 3t, yellow amorphous powder, 41% yield; ^1^H NMR (600 MHz, CDCl_3_) *δ*: 8.06–8.05 (m, 3H), 7.94 (s, 1H), 7.69 (d, *J* = 7.8 Hz, 1H), 7.42 (td, *J* = 7.2, 1.2 Hz, 1H), 7.34 (t, *J* = 7.2 Hz, 1H), 7.29 (t, *J* = 7.2 Hz, 1H), 7.22–7.18 (m, 2H), 6.88 (d, *J* = 8.0 Hz, 1H), 5.70 (s, 1H), 4.97 (ddd, *J* = 12.6, 4.8, 1.8 Hz, 1H), 3.26 (td, *J* = 11.4, 4.2 Hz, 1H), 3.12–3.09 (m, 1H), 3.03–2.97 (m, 1H), and 2.47 (s, 3H); ^13^C NMR (150 MHz, CDCl_3_) *δ*: 164.5, 150.3, 139.5, 138.6, 133.2, 133.1 (q, *J* = 35 Hz), 129.6, 129.0, 128.2 (d, *J* = 3 Hz), 126.6, 124.8, 124.5, 124.2 (d, *J* = 3 Hz), 123.9 (d, *J* = 270 Hz), 123.02, 122.96, 121.7, 121.6 (d, *J* = 4.5 Hz), 119.6, 117.1, 110.4, 68.3, 39.4, 36.6, and 20.6; HRESIMS (*m*/*z*): 516.1523 [M + H]^+^ (calcd for C_27_H_20_F_6_N_3_O, 516.1511).

Compound 3u, yellow amorphous powder, 89% yield; ^1^H NMR (400 MHz, CDCl_3_) *δ*: 8.99 (dd, *J* = 4.4, 1.6 Hz, 1H), 8.24 (d, *J* = 9.2 Hz, 1H), 8.17 (d, *J* = 8.4 Hz, 1H), 8.02 (dd, *J* = 7.8, 1.6 Hz, 1H), 7.92–7.81 (m, 2H), 7.71–7.67 (m, 1H), 7.50 (dd, *J* = 8.4, 4.4 Hz, 1H), 7.29–7.27 (m, 3H), 7.26–7.24 (m, 1H), 7.08 (t, *J* = 7.6 Hz, 1H), 6.64 (d, *J* = 8.0 Hz, 1H), 5.92 (s, 1H), 4.98 (ddd, *J* = 12.6, 4.8, 1.8 Hz, 1H), 3.33 (td, *J* = 11.4, 4.2 Hz, 1H), 3.15–3.10 (m, 1H), 3.06–2.98 (m, 1H), and 2.40 (s, 3H); ^13^C NMR (150 MHz, CDCl_3_) *δ*: 164.7, 151.2, 150.5, 147.4, 139.0, 136.2, 135.8, 132.9, 131.0, 129.7, 129.2, 128.8, 128.5, 126.2, 125.4, 123.8 × 2, 123.7, 122.3, 122.0, 120.9, 119.2, 115.8, 110.8, 68.1, 39.4, 36.5, and 20.7; HRESIMS (*m*/*z*): 431.1872 [M + H]^+^ (calcd for C_28_H_23_N_4_O, 431.1869).

Compound 3v, yellow amorphous powder, 81% yield; ^1^H NMR (400 MHz, CDCl_3_) *δ*: 8.08 (dd, *J* = 7.8, 1.6 Hz, 1H), 7.65 (d, *J* = 6.8 Hz, 1H), 7.45–7.27 (m, 5H), 7.25–7.21 (m, 2H), 7.15 (td, *J* = 7.6, 1.2 Hz, 1H), 6.91 (d, *J* = 8.0 Hz, 1H), 5.85 (s, 1H), 4.95 (ddd, *J* = 12.6, 4.8, 1.8 Hz, 1H), 3.27 (td, *J* = 11.4, 4.2 Hz, 1H), 3.11–3.05 (m, 1H), 3.02–2.91 (m, 1H), and 2.43 (s, 3H); ^13^C NMR (150 MHz, CDCl_3_) *δ*: 164.7, 150.6, 138.9, 135.6, 133.0, 129.5, 128.9, 126.0, 125.9, 125.7, 123.7, 123.67, 123.60, 122.2, 120.7, 120.1, 119.0, 115.3, 111.2, 68.0, 39.5, 36.3, and 20.6; HRESIMS (*m*/*z*): 386.1327 [M + H]^+^ (calcd for C_23_H_20_N_3_OS, 386.1316).

### *In vitro* cytotoxicity assay

HCT-116 (human colorectal adenocarcinoma cells), 4T1 (murine breast cancer cells), and SU-DHL-6 (human lymphoma cells) were purchased from the American Type Culture Collection (ATCC, Manassas, VA, USA). The antiproliferative activity of the compounds against cancer cell lines was measured by an MTT assay. MTT (3-(4,5-dimethylthiazol-2-yl)-2,5-diphenyltetrazolium bromide) was purchased from Sigma-Aldrich (St. Louis, MO, USA). Cells were seeded in 96-well plates at a density of 5 × 10^3^ cells per well and maintained in a humidified atmosphere with 5% CO_2_ at 37 °C. After overnight incubation, the cells were treated with the test compounds for 48 h. Then, 20 µL of MTT solution (5 mg mL^−1^) was added to each well, and the plates were incubated for an additional 4 h. After removal of the medium, the insoluble formazan crystals were solubilized using 150 µL of DMSO per well. Absorbance was measured at 490 nm using a SpectraMax CMax Plus microplate reader (Molecular Devices, Sunnyvale, CA, USA). All experiments were performed in triplicate.

## Author contributions

Data curation, visualization: Jing Wang, Yang Yang, and Ming-Li Zhou; investigation: Yang Yang, and Ming-Li Zhou; writing – original draft: Jing Wang; writing – review & editing: Jin-Bu Xu, and Lian Sun; project administration, funding acquisition: Feng Gao. Finally, all authors revised and approved the final version of the manuscript.

## Conflicts of interest

The authors declare no conflict of interest.

## Supplementary Material

RA-016-D5RA09118G-s001

## Data Availability

The data underlying this study are available in the published article and its supplementary information (SI). Supplementary information: spectroscopic data (^1^H and ^13^C NMR). See DOI: https://doi.org/10.1039/d5ra09118g.

## References

[cit1] Guo Z. R. (2017). Acta Pharm. Sin. B.

[cit2] Chopra B., Dhingra A. K. (2021). Phytother. Res..

[cit3] Iwaoka E., Wang S., Matsuyoshi N., Kogure Y., Aoki S., Yamamoto S., Noguchi K., Dai Y. (2016). J. Nat. Med..

[cit4] Wu P. P., Chen Y. (2019). Hum. Cell.

[cit5] Ko H. C., Wang Y. H., Liou K. T., Chen C. M., Chen C. H., Wang W. Y., Chang S., Hou Y. C., Chen K. T., Chen C. F., Shen Y. C. (2007). Eur. J. Pharmacol..

[cit6] Wu J. Y., Chang M. C., Chen C. S., Lin H. C., Tsai H. P., Yang C. C., Yang C. H., Lin C. M. (2013). Planta Med..

[cit7] Fang Z. L., Tang Y. Q., Ying J. M., Tang C. L., Wang Q. W. (2020). Chin. Med..

[cit8] Panda M., Tripathi S. K., Zengin G., Biswal B. K. (2023). Cell Biol. Toxicol..

[cit9] Sun Q., Xie L., Song J. W., Li X. F. (2020). J. Ethnopharmacol..

[cit10] Dong G. Q., Sheng C. Q., Wang S. Z., Miao Z. Y., Yao J. Z., Zhang W. M. (2010). J. Med. Chem..

[cit11] Dong G. Q., Wang S. Z., Miao Z. Y., Yao J. Z., Zhang Y. Q., Guo Z. Z., Zhang W. N., Sheng C. Q. (2012). J. Med. Chem..

[cit12] Xu S. T., Yao H., Qiu Y. Y., Zhou M. Z., Li D. H., Wu L., Yang D. H., Chen Z. S., Xu J. Y. (2021). J. Med. Chem..

[cit13] Lei F., Xiong Y. X., Wang Y. Q., Zhang H. H., Liang Z. Y., Li J. F., Feng Y. Y., Hao X. Y., Wang Z. (2022). J. Med. Chem..

[cit14] Wang Z., Xiong Y. X., Peng Y., Zhang X., Li S., Peng Y., Peng X., Zhuo L. S., Jiang W. F. (2023). Eur. J. Med. Chem..

[cit15] Wang S. Z., Fang K., Dong G. Q., Chen S. Q., Liu N., Miao Z. Y., Yao J. Z., Li J., Zhang W. N., Sheng C. Q. (2015). J. Med. Chem..

[cit16] Chen S. T., Zhang X., Mo H. X., Peng Y., An Z. G., Wu J. B., Wei X. Z., Zhang S. Y., Xiong Y. X., Jiang W. F., Peng X., Zhuo L. S., Lei Z. W., Wang Z., Hu Z. C. (2024). Eur. J. Med. Chem..

[cit17] Liang C. Y., Xia J., Song H. H., Zhou Z. G., Xue Y., Yao Q. Z. (2014). J. Chem. Pharm. Res..

[cit18] Hong B. K., Luo T. P., Lei X. G. (2020). ACS Cent. Sci..

[cit19] Liu J., Yang Y., Ouyang K. B., Zhang W. X. (2021). Green Synth. Catal..

[cit20] Rago A. J., Dong G. B. (2021). Green Synth. Catal..

[cit21] Zhou M. L., Liu N. L., Sun L., Li X. H., Xu J. B., Gao F. (2025). RSC Med. Chem..

[cit22] Sun L., Li X. L., Huang Q. S., Ji W. S., Li X. H., Xu J. B., Gao F. (2025). J. Nat. Prod..

[cit23] Tang Q. H., Lu Y. G., Song J. Y., He Z. Y., Xu J. B., Tan J., Gao F., Li X. H. (2024). Fitoterapia.

[cit24] Wang K., Fan R. Q., Wei X., Fang W. W. (2022). Green Synth. Catal..

[cit25] Dhakshinamoorthy A., Asiri A. M., Garcia H. (2015). Chem. Soc. Rev..

[cit26] Chen H., Lei M., Hu L. H. (2014). Tetrahedron.

[cit27] Xie Y. X., Pi S. F., Wang J., Yin L., Li J. H. (2006). J. Org. Chem..

