# Supplementary material for: Cu-catalyzed late-stage diversification and anti-proliferative activity evaluation of evodiamine
Source: RSC Adv. 2026 Mar 10;16(14):13000–6. doi: 10.1039/d5ra09118g (PMC12973284; doi:10.1039/d5ra09118g)
Supplement: RA-016-D5RA09118G-s001 [file RA-016-D5RA09118G-s001.pdf]

Supplementary Information (SI) for RSC Advances.
This journal is © The Royal Society of Chemistry 2026

Cu-catalyzed late-stage diversification and anti-proliferative activity evaluation of evodiamine

Jing Wang, Yang Yang, Ming-Li Zhou, Feng Gao,* Jin-Bu Xu,* Lian Sun*

Sichuan Engineering Research Center for Biomimetic Synthesis of Natural Drugs, School of Life Science and Engineering, Southwest Jiaotong University, Chengdu 610031, People's Republic of China

*** Corresponding Author:**

Prof. Feng Gao

E-mail: gaof@swjtu.edu.cn

Dr. Jin-Bu Xu

E-mail: xujinbu@swjtu.edu.cn

Dr. Lian Sun

E-mail: liansun@swjtu.edu.cn

Content

Figure 1. ^{1}H NMR spectrum of compound **3a** (600 MHz, $CDCl_3$) S3
Figure 2. ^{13}C NMR spectrum of compound **2** (100 MHz, $CDCl_3$) S3
Figure 3. ^{1}H NMR spectrum of compound **3b** (600 MHz, $CDCl_3$) S4
Figure 5. ^{1}H NMR spectrum of compound **3c** (600 MHz, $CDCl_3$) S5
Figure 6. ^{13}C NMR spectrum of compound **3c** (100 MHz, $CDCl_3$) S5
Figure 7. ^{1}H NMR spectrum of compound **3d** (400 MHz, $CDCl_3$) S6
Figure 8. ^{13}C NMR spectrum of compound **3d** (150 MHz, $CDCl_3$) S6
Figure 9. ^{1}H NMR spectrum of compound **3e** (600 MHz, $CDCl_3$) S7
Figure 10. ^{13}C NMR spectrum of compound **3e** (150 MHz, $CDCl_3$) S7
Figure 11. ^{1}H NMR spectrum of compound **3f** (600 MHz, $CDCl_3$) S8
Figure 12. ^{13}C NMR spectrum of compound **3f** (150 MHz, $CDCl_3$) S8
Figure 13. ^{1}H NMR spectrum of compound **3g** (600 MHz, $CDCl_3$) S9
Figure 14. ^{13}C NMR spectrum of compound **3g** (100 MHz, $CDCl_3$) S9
Figure 15. ^{1}H NMR spectrum of compound **3h** (600 MHz, CD_3OD) S10
Figure 16. ^{13}C NMR spectrum of compound **3h** (150 MHz, $CDCl_3$) S10
Figure 17. ^{1}H NMR spectrum of compound **3i** (600 MHz, $CDCl_3$) S11
Figure 19. ^{1}H NMR spectrum of compound **3j** (600 MHz, $CDCl_3$) S12
Figure 21. ^{1}H NMR spectrum of compound **3k** (600 MHz, $CDCl_3$) S13
Figure 23. ^{1}H NMR spectrum of compound **3l** (600 MHz, $CDCl_3$) S14
Figure 25. ^{1}H NMR spectrum of compound **3m** (600 MHz, $CDCl_3$) S15
Figure 27. ^{1}H NMR spectrum of compound **3n** (400 MHz, $CDCl_3$) S16
Figure 29. ^{1}H NMR spectrum of compound **3o** (600 MHz, $CDCl_3$) S17
Figure 31. ^{1}H NMR spectrum of compound **3p** (600 MHz, $CDCl_3$) S18
Figure 33. ^{1}H NMR spectrum of compound **3q** (600 MHz, $CDCl_3$) S19
Figure 34. ^{13}C NMR spectrum of compound **3q** (150 MHz, $CDCl_3$) S19
Figure 35. ^{1}H NMR spectrum of compound **3r** (400 MHz, $CDCl_3$) S20
Figure 36. ^{13}C NMR spectrum of compound **3r** (150 MHz, $CDCl_3$) S20
Figure 37. ^{1}H NMR spectrum of compound **3s** (400 MHz, $CDCl_3$) S21
Figure 38. ^{13}C NMR spectrum of compound **3s** (150 MHz, $CDCl_3$) S21
Figure 39. ^{1}H NMR spectrum of compound **3t** (600 MHz, $CDCl_3$) S22
Figure 40. ^{13}C NMR spectrum of compound **3t** (150 MHz, $CDCl_3$) S22
Figure 43. ^{1}H NMR spectrum of compound **3v** (400 MHz, $CDCl_3$) S24
Figure 44. ^{13}C NMR spectrum of compound **3v** (150 MHz, $CDCl_3$) S24

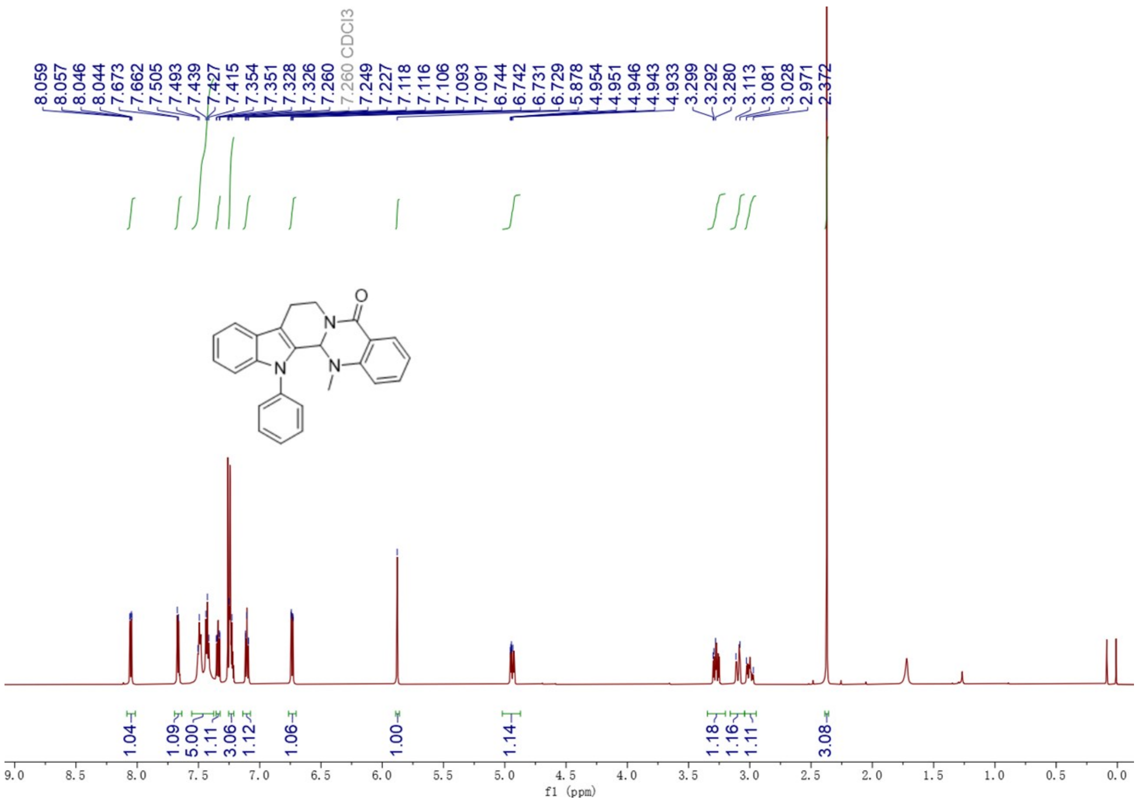

Figure 1. ^{1}H NMR spectrum of compound **3a** (600 MHz, $CDCl_3$)

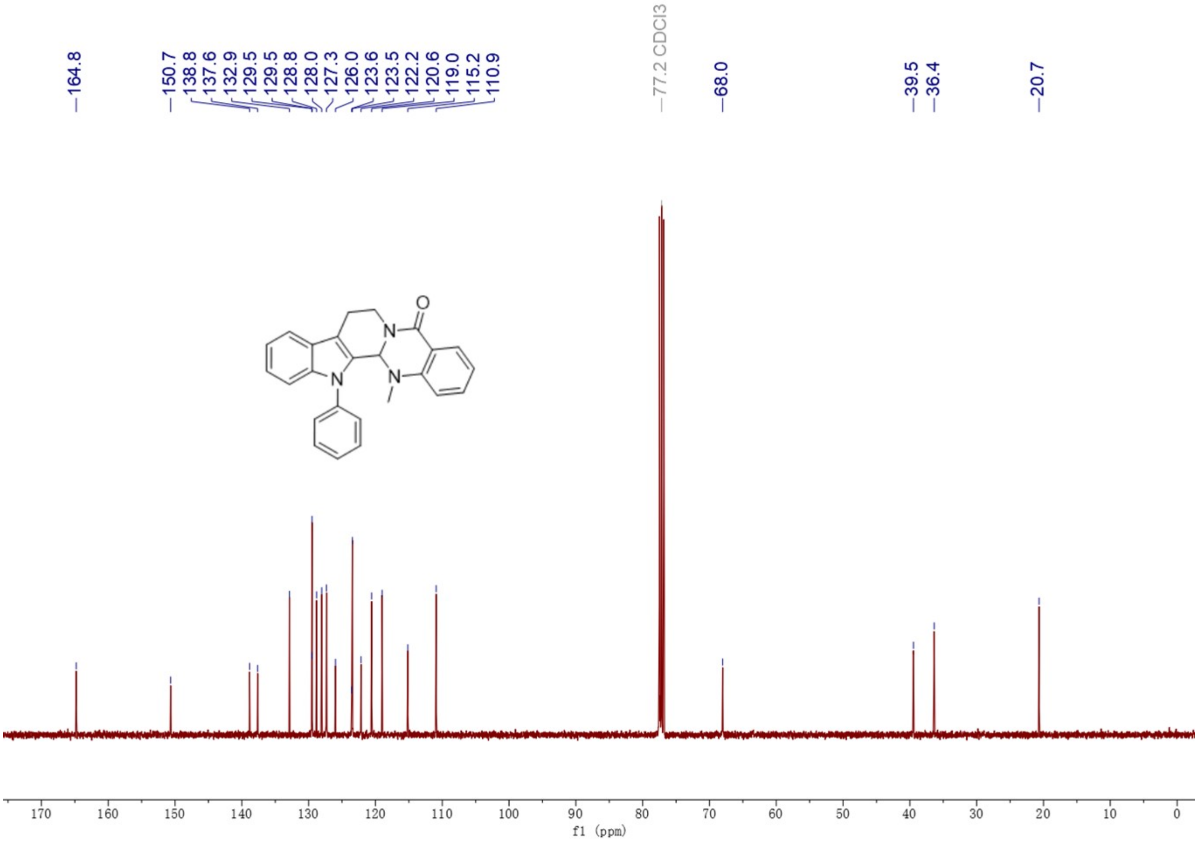

Figure 2. ^{13}C NMR spectrum of compound **2** (100 MHz, $CDCl_3$)

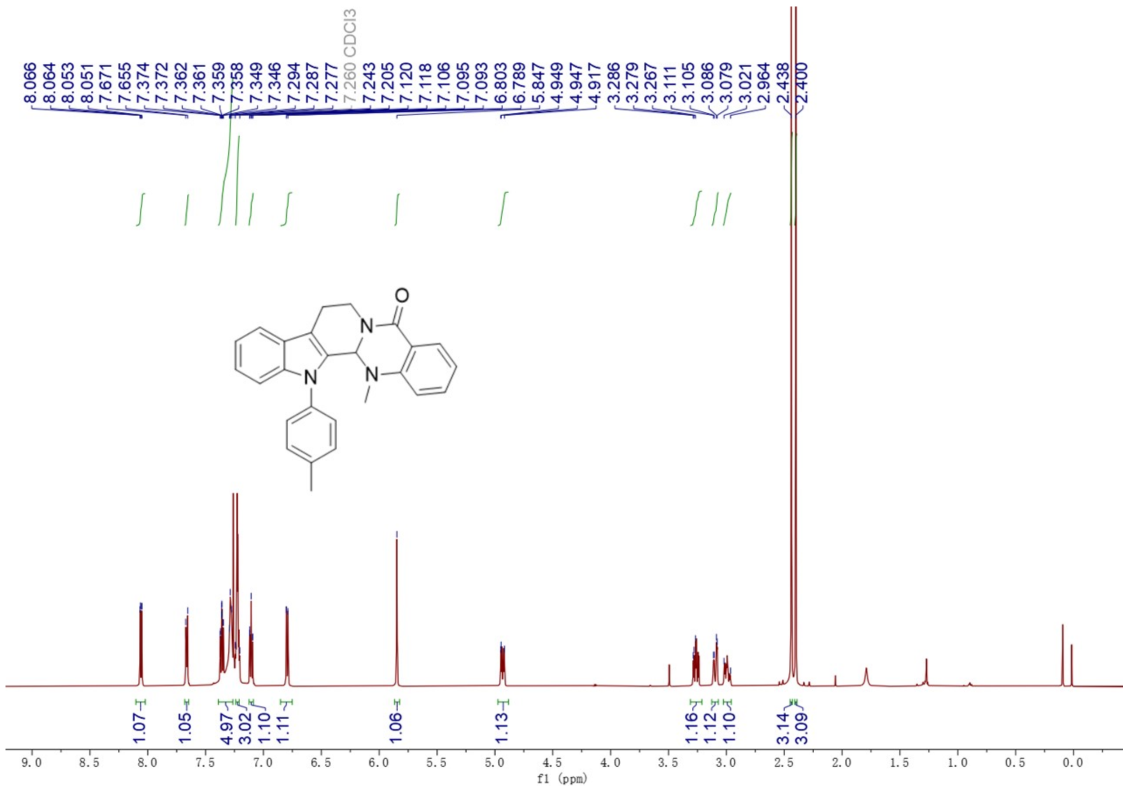

Figure 3. ^{1}H NMR spectrum of compound **3b** (600 MHz, $CDCl_3$)

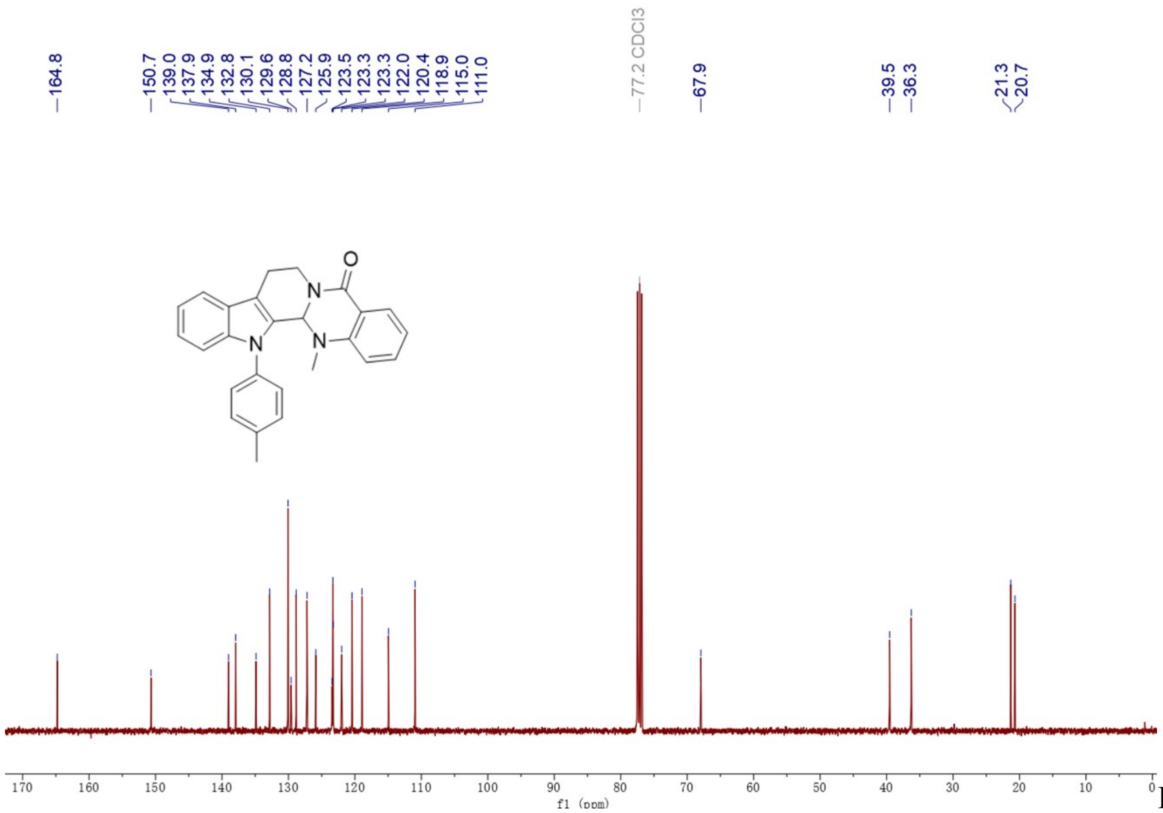

Fig

ure 4. ^{13}C NMR spectrum of compound **3b** (100 MHz, $CDCl_3$)

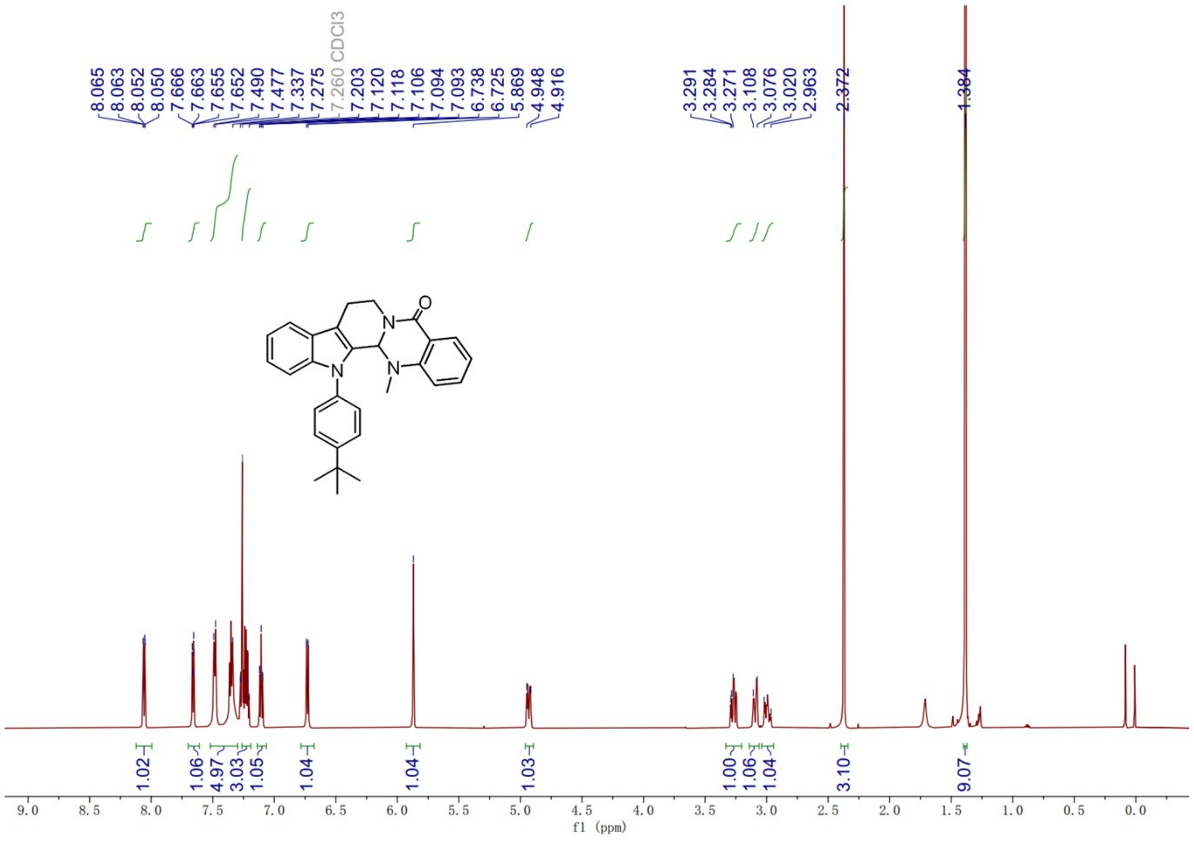

Figure 5. ^{1}H NMR spectrum of compound **3c** (600 MHz, $CDCl_3$)

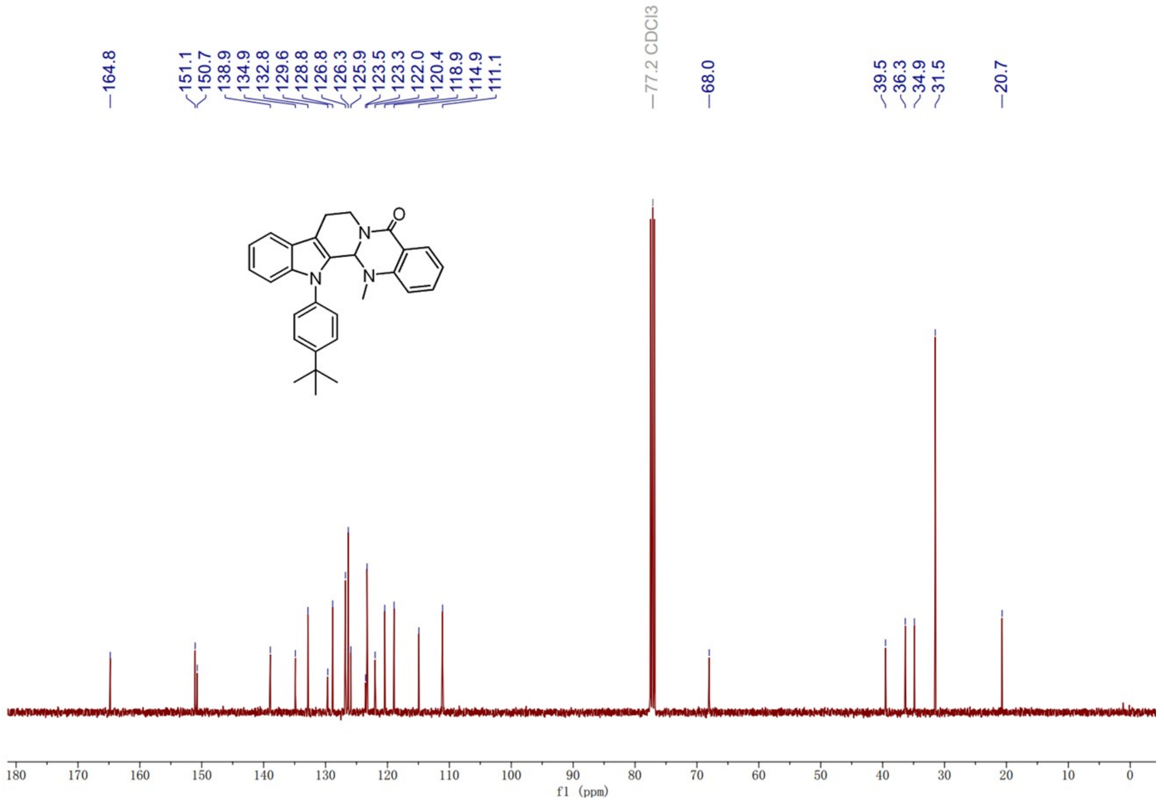

Figure 6. ^{13}C NMR spectrum of compound **3c** (100 MHz, $CDCl_3$)

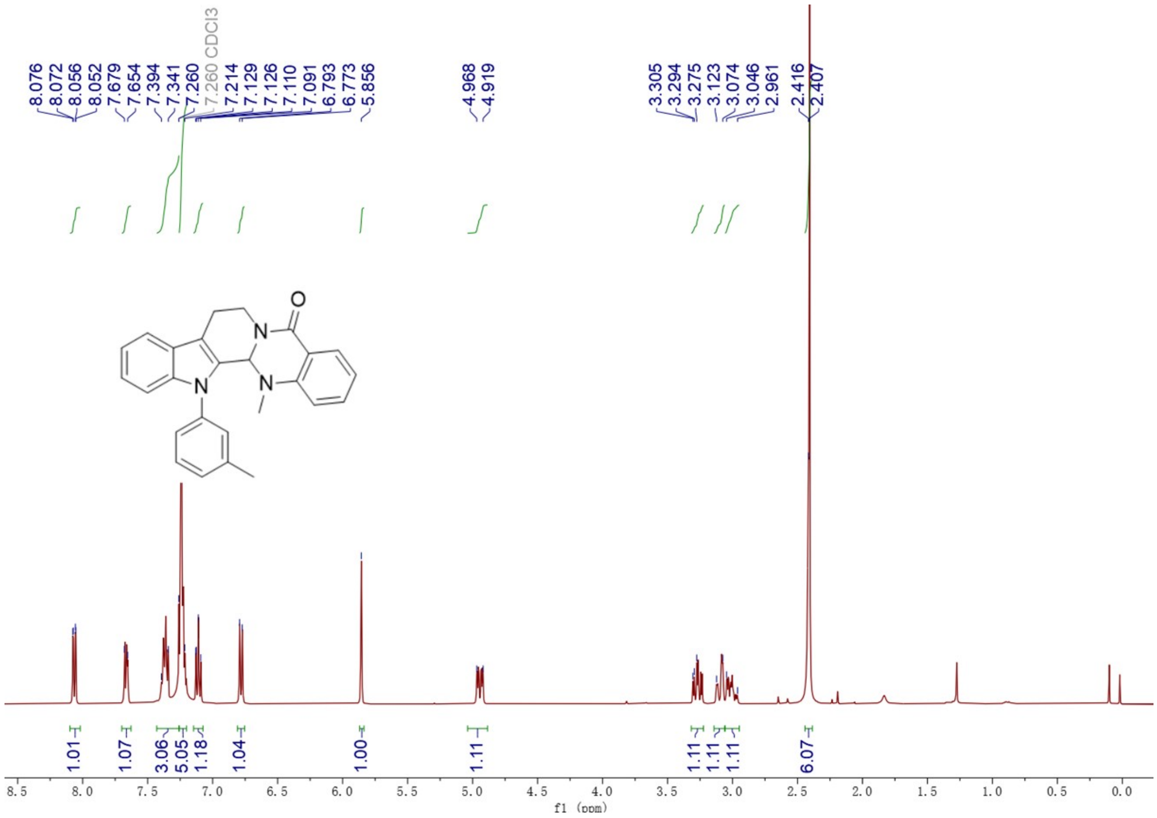

Figure 7. ^{1}H NMR spectrum of compound **3d** (400 MHz, $CDCl_3$)

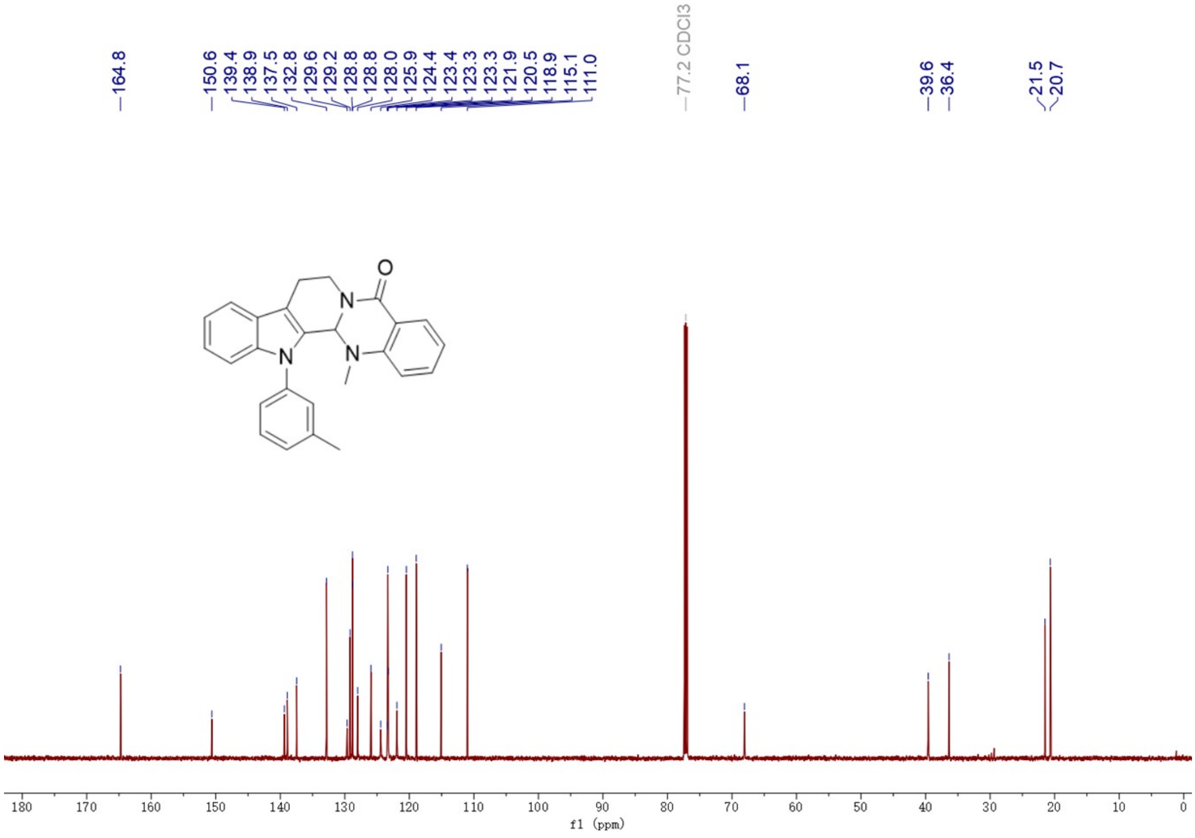

Figure 8. ^{13}C NMR spectrum of compound **3d** (150 MHz, $CDCl_3$)

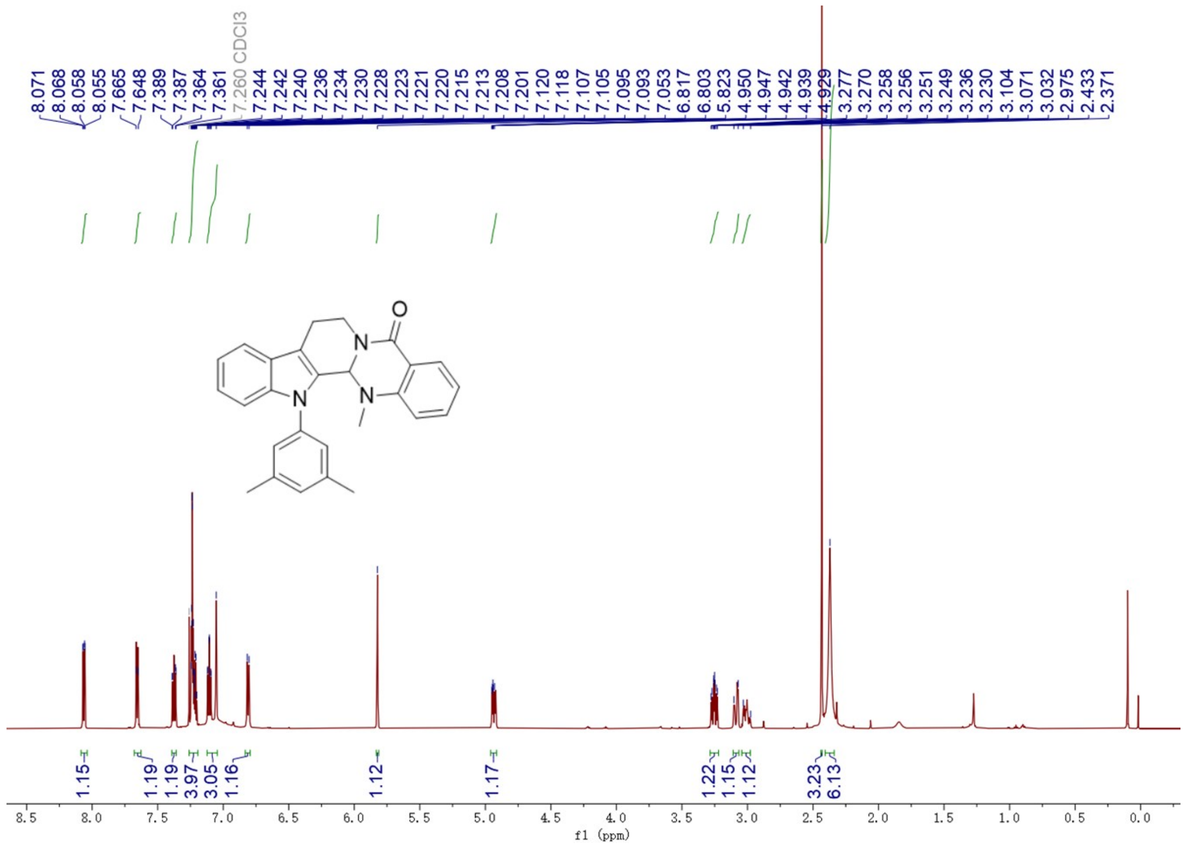

Figure 9. ^{1}H NMR spectrum of compound **3e** (600 MHz, $CDCl_3$)

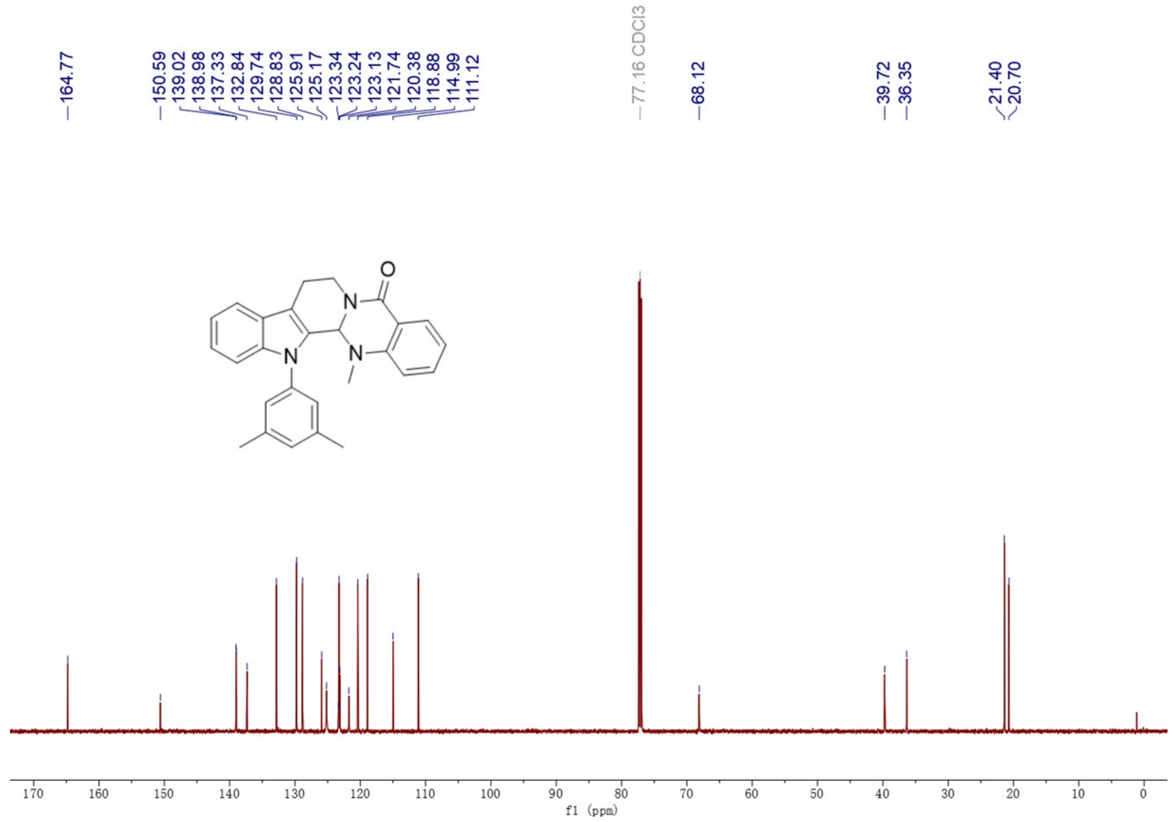

Figure 10. ^{13}C NMR spectrum of compound **3e** (150 MHz, $CDCl_3$)

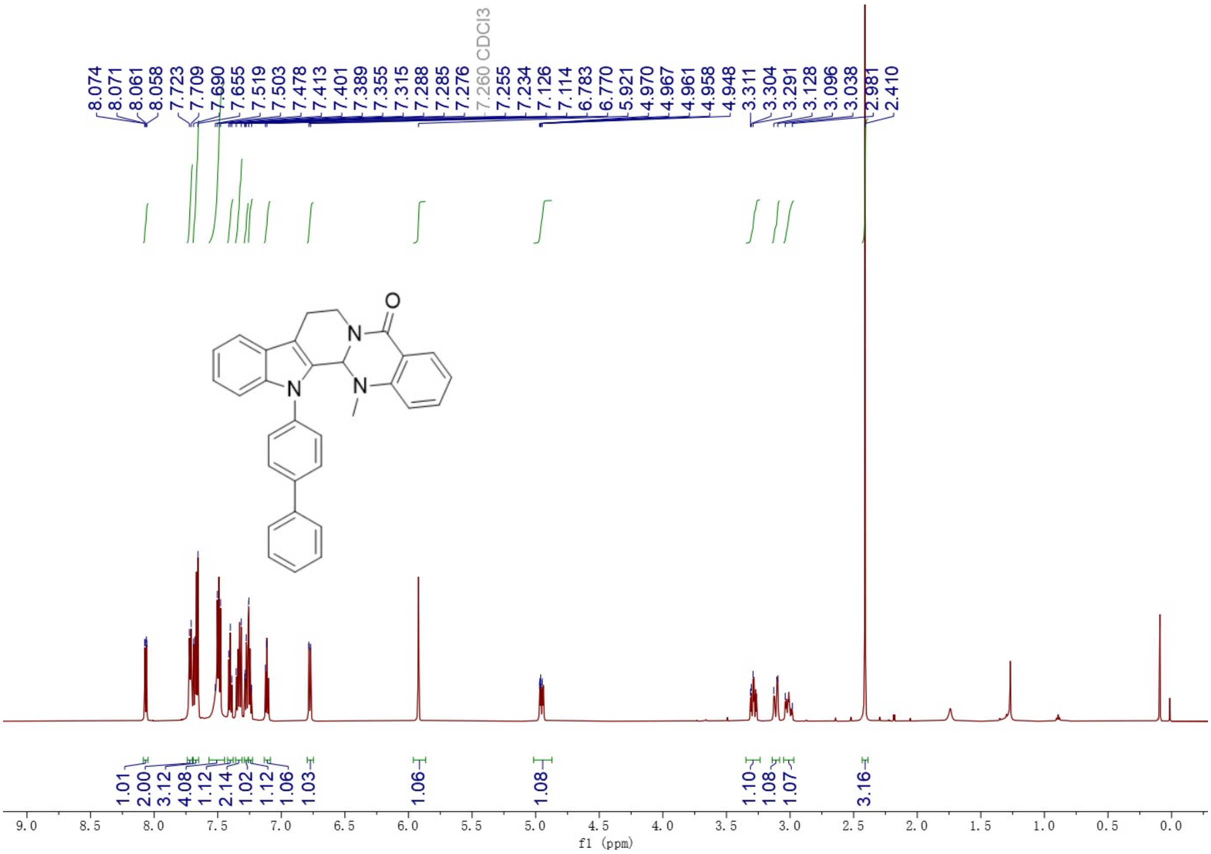

Figure 11. ^{1}H NMR spectrum of compound **3f** (600 MHz, $CDCl_3$)

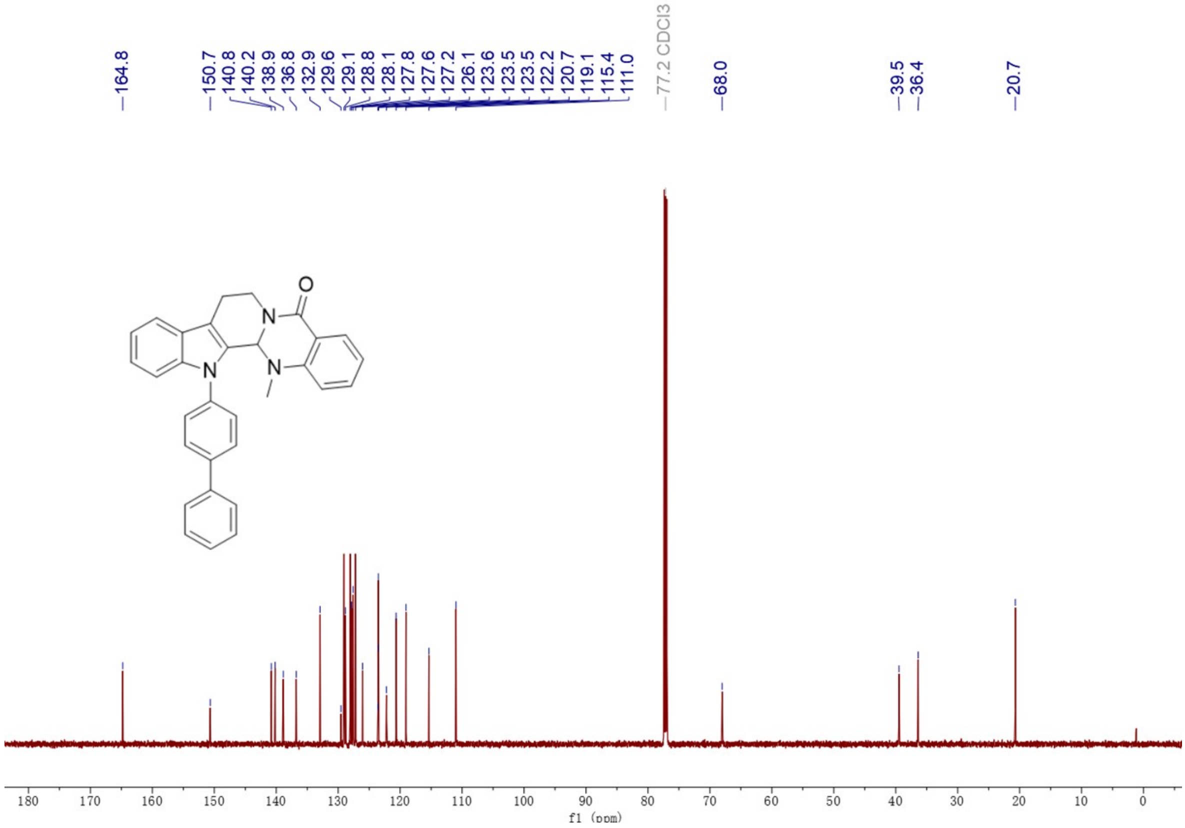

Figure 12. ^{13}C NMR spectrum of compound **3f** (150 MHz, $CDCl_3$)

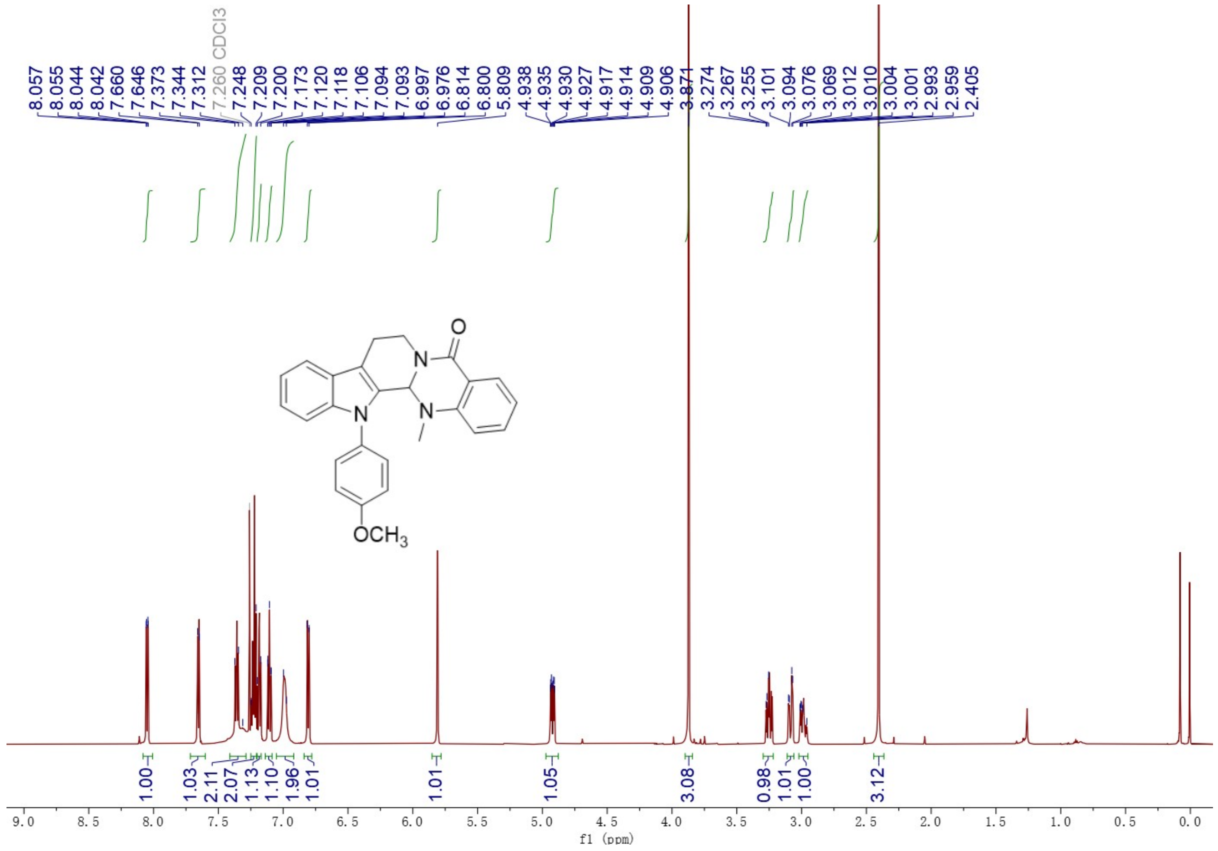

Figure 13. ^{1}H NMR spectrum of compound **3g** (600 MHz, $CDCl_3$)

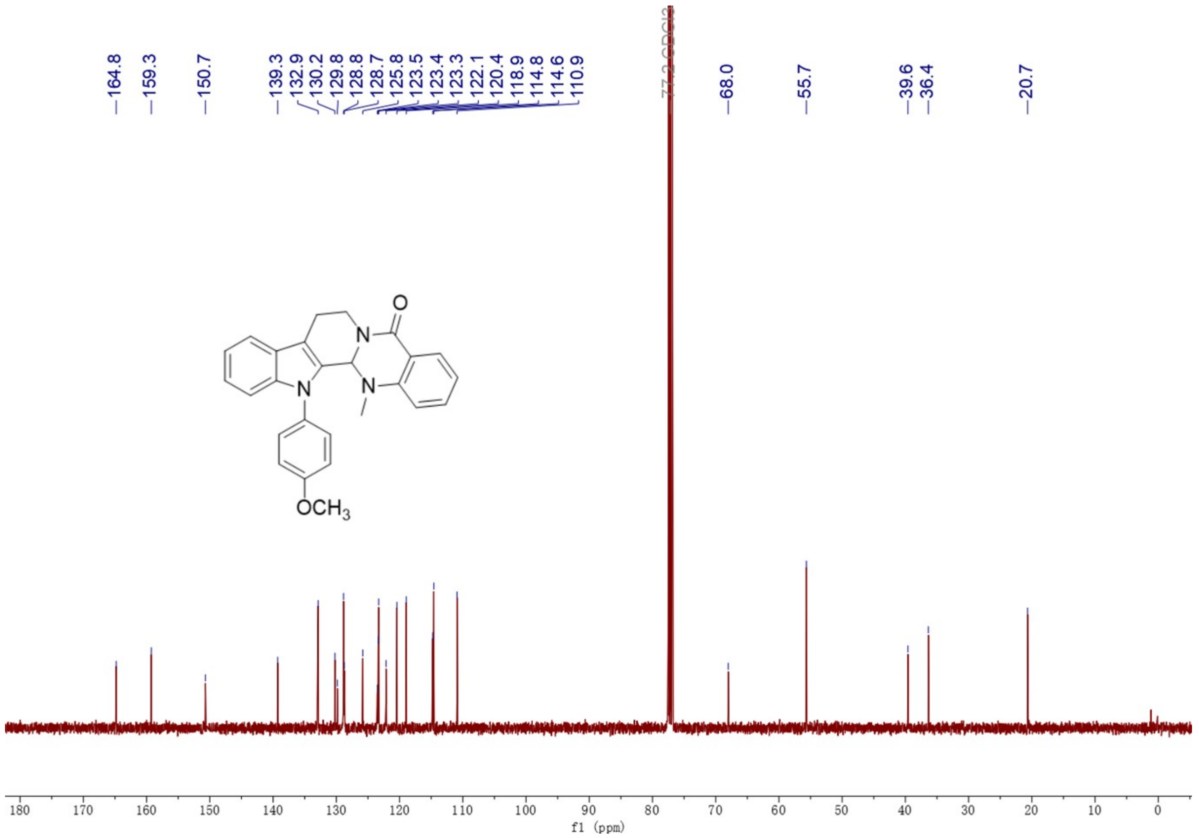

Figure 14. ^{13}C NMR spectrum of compound **3g** (100 MHz, $CDCl_3$)

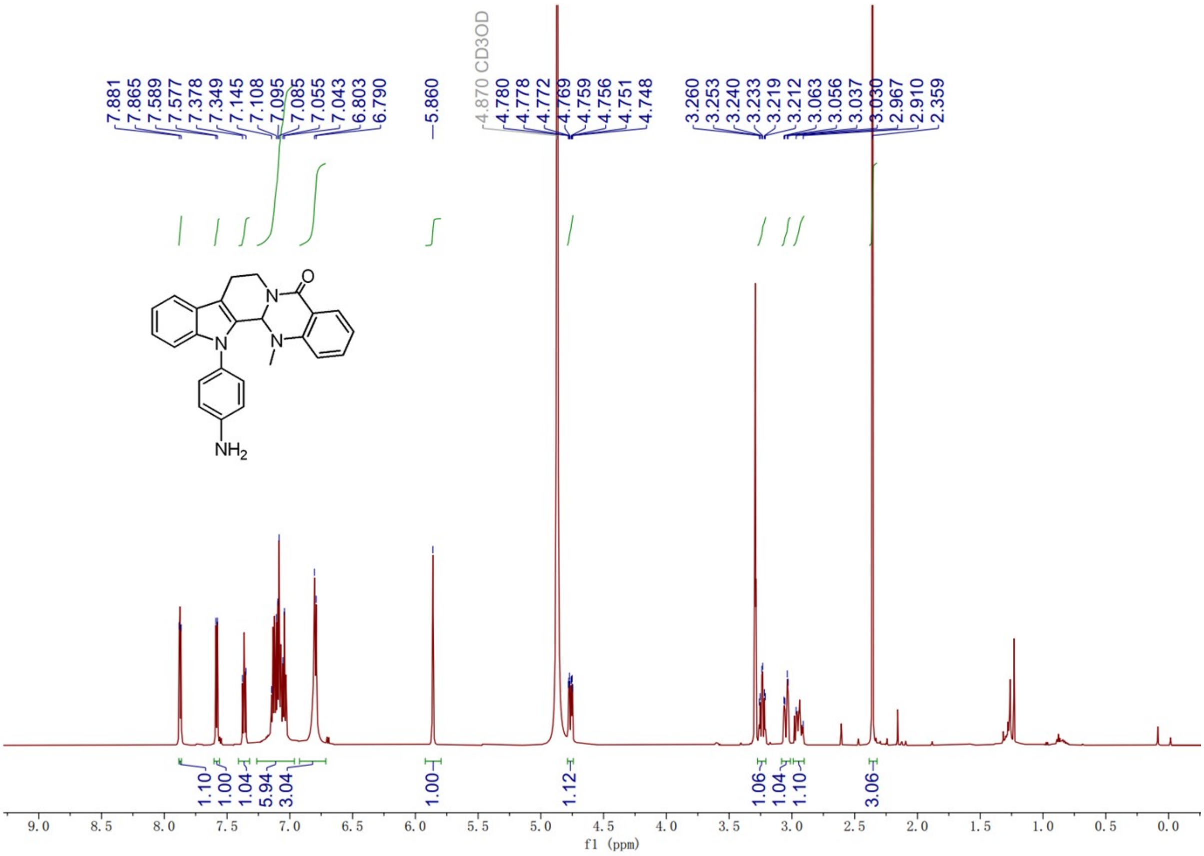

Figure 15. ^{1}H NMR spectrum of compound **3h** (600 MHz, CD_3OD)

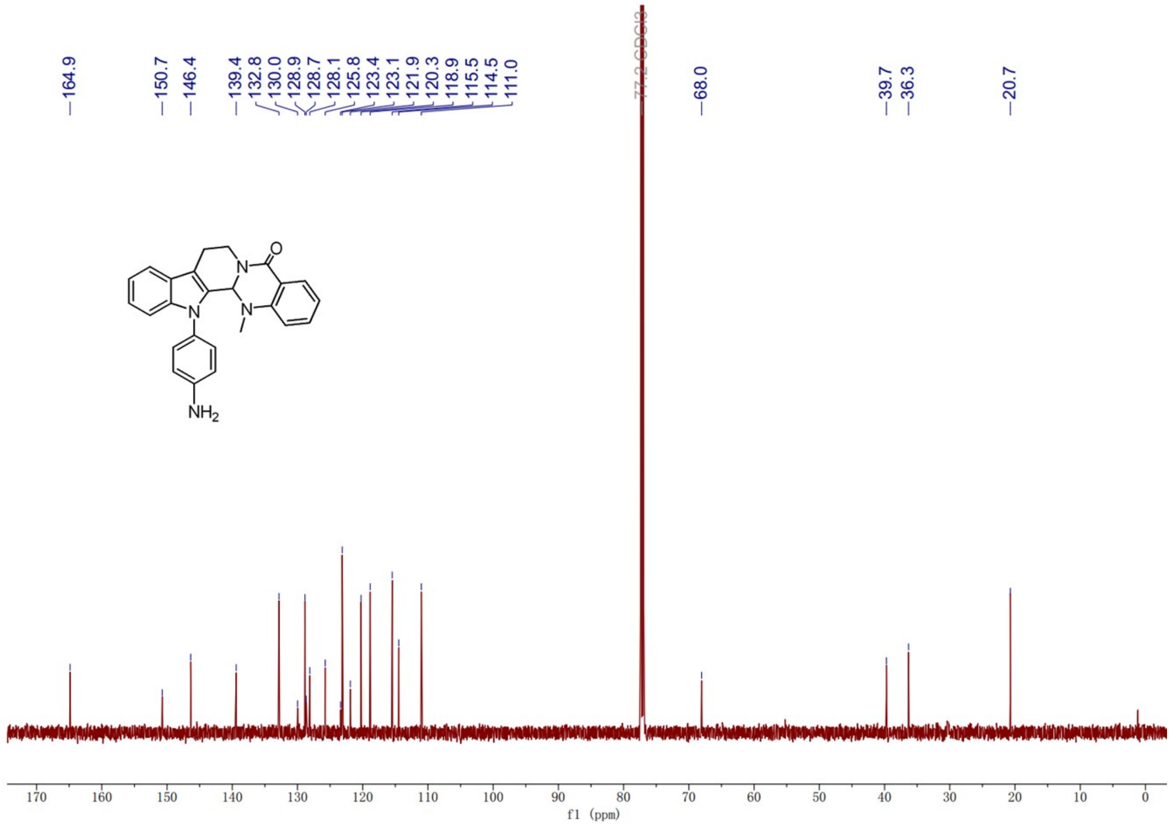

Figure 16. ^{13}C NMR spectrum of compound **3h** (150 MHz, $CDCl_3$)

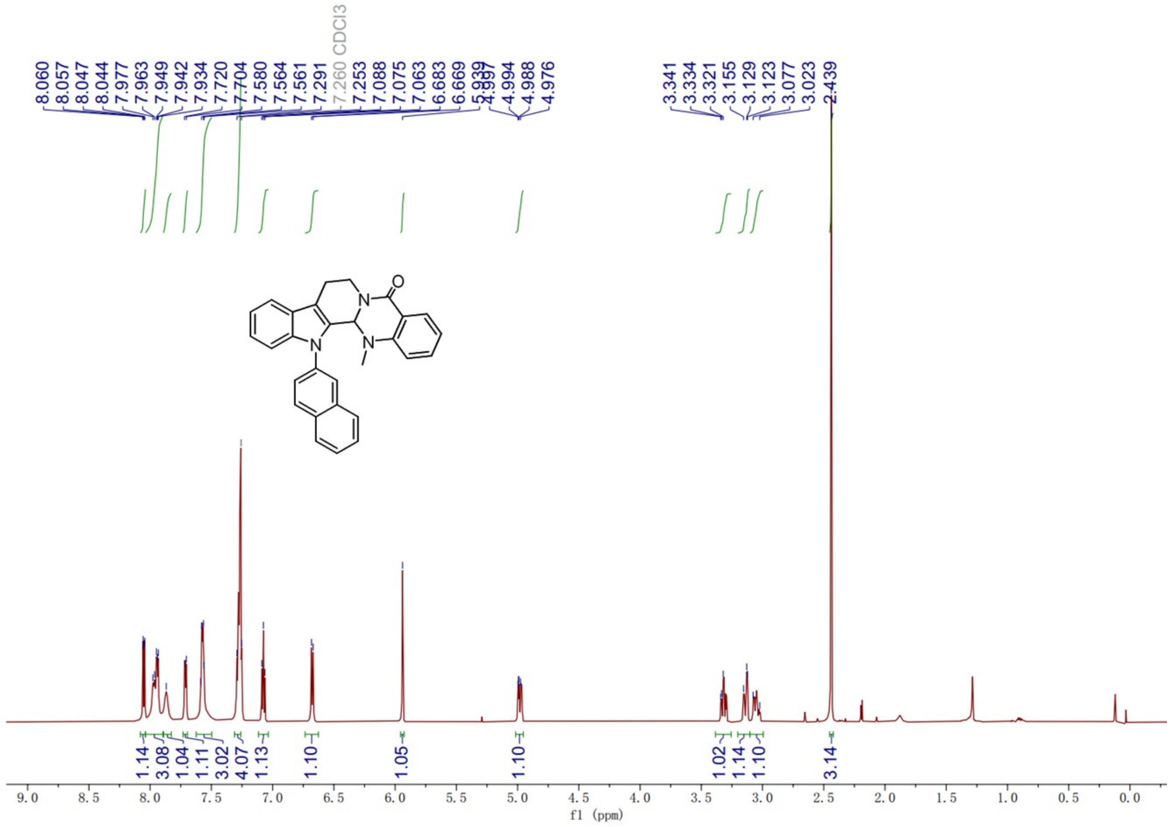

Figure 17. ^{1}H NMR spectrum of compound **3i** (600 MHz, $CDCl_3$)

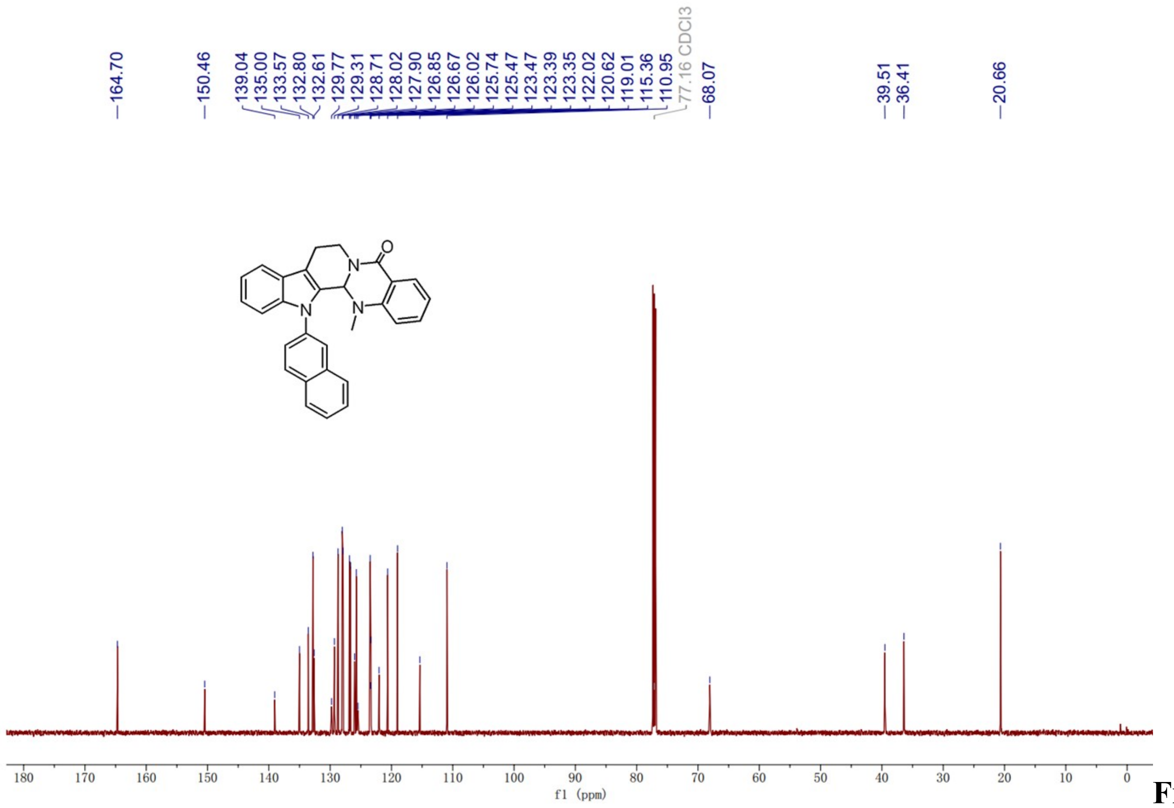

Fig

ure 18. ^{13}C NMR spectrum of compound **3i** (150 MHz, $CDCl_3$)

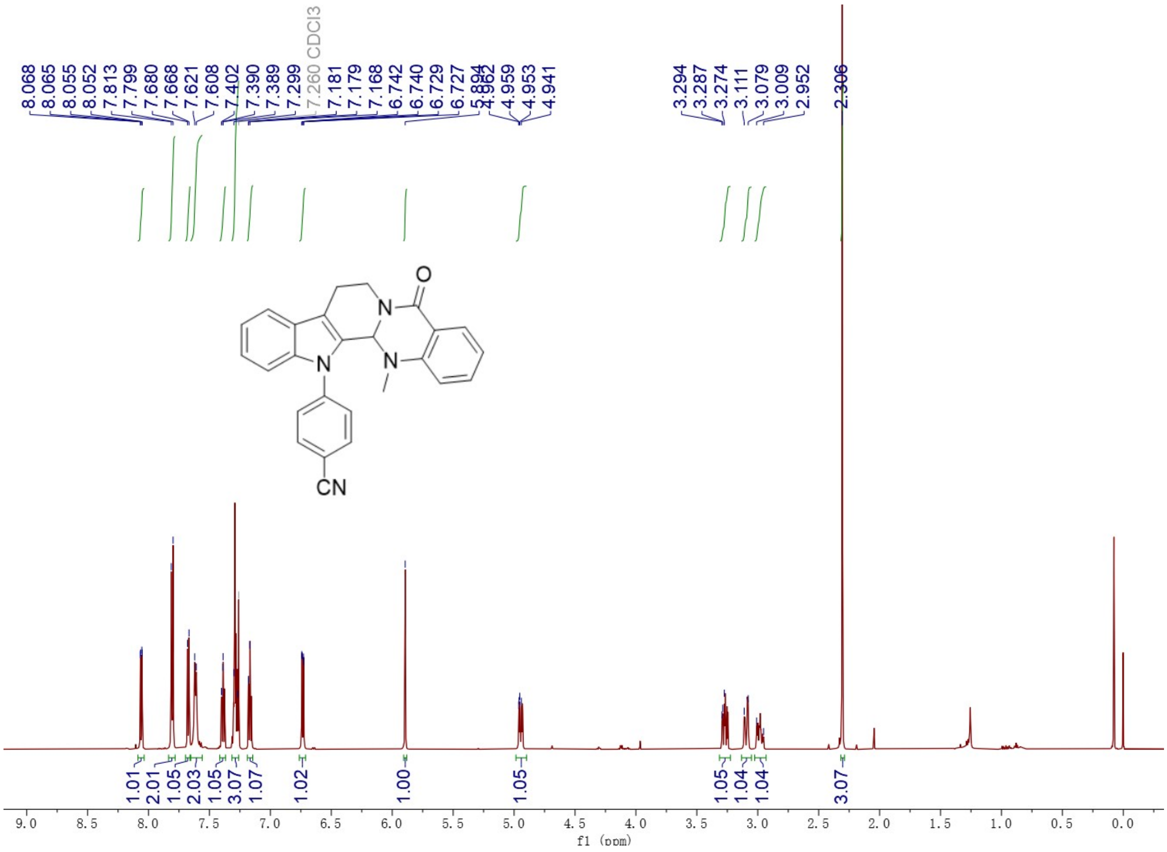

Figure 19. ^{1}H NMR spectrum of compound **3j** (600 MHz, $CDCl_3$)

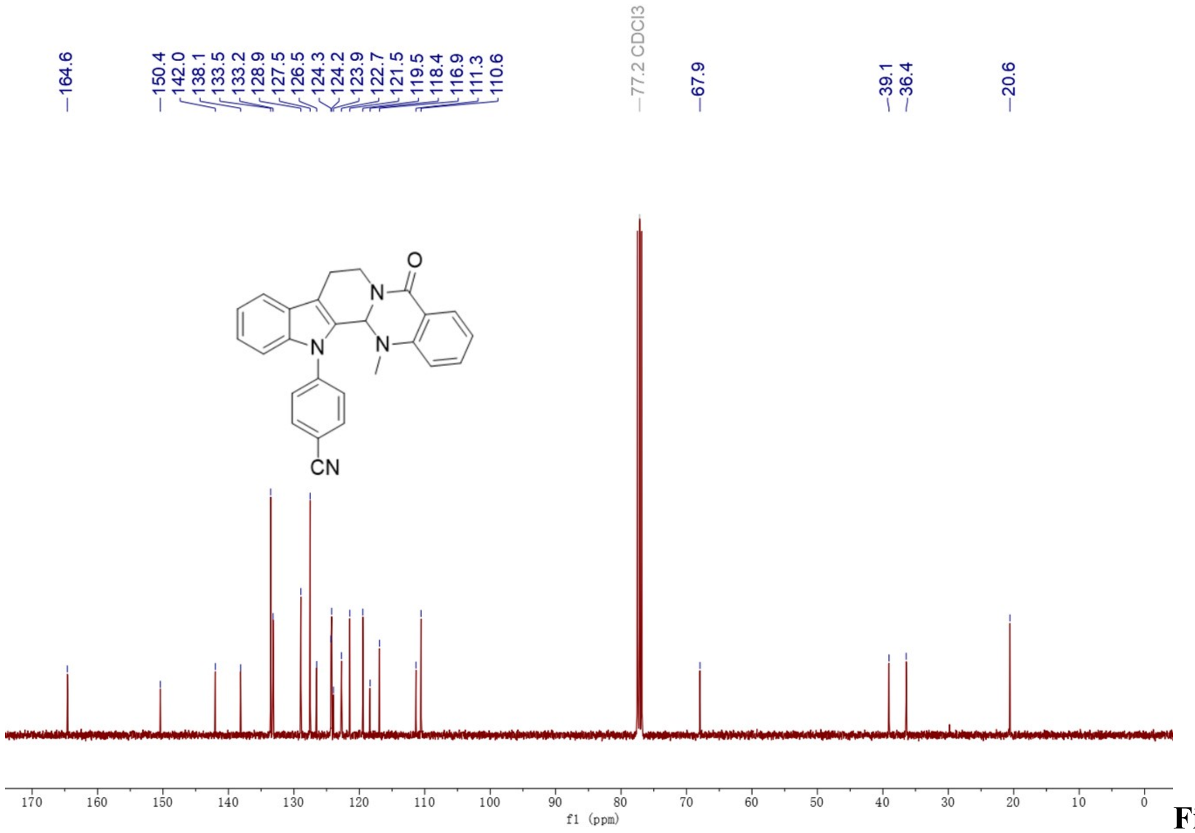

Figure 20. ^{13}C NMR spectrum of compound **3j** (100 MHz, $CDCl_3$)

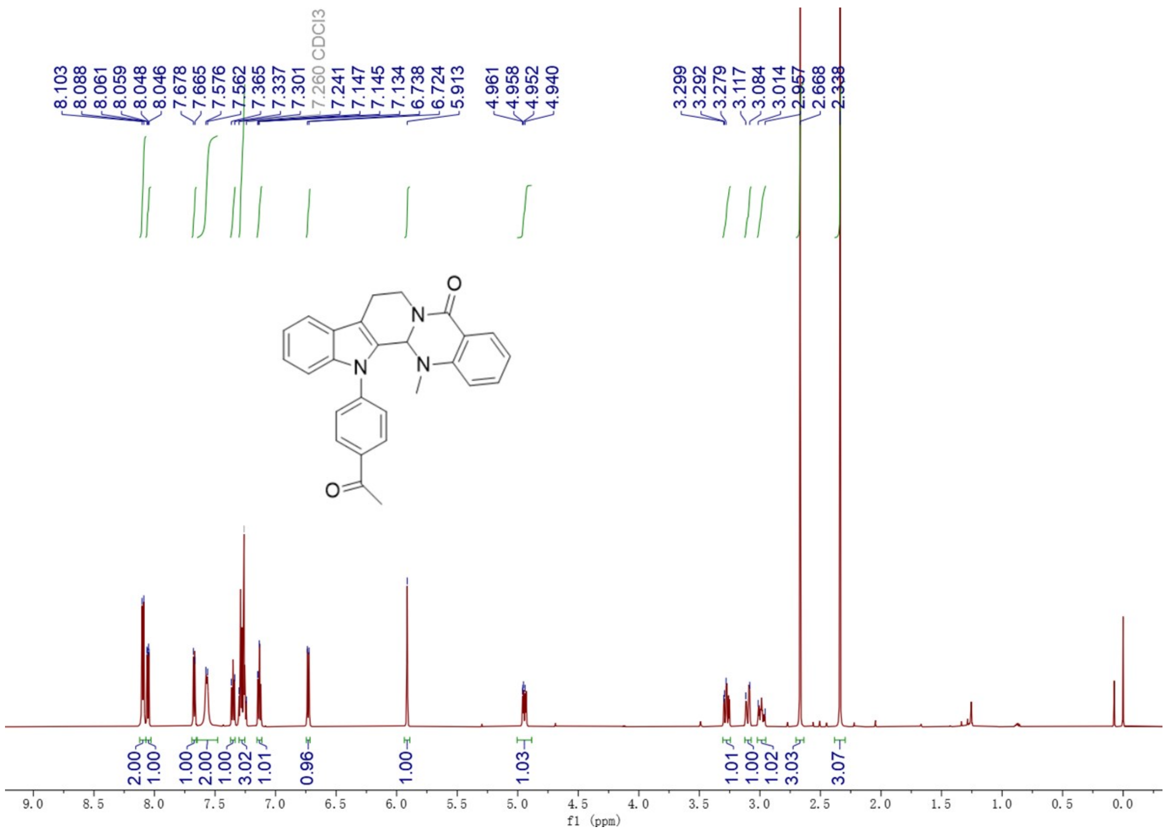

Figure 21. ^{1}H NMR spectrum of compound **3k** (600 MHz, $CDCl_3$)

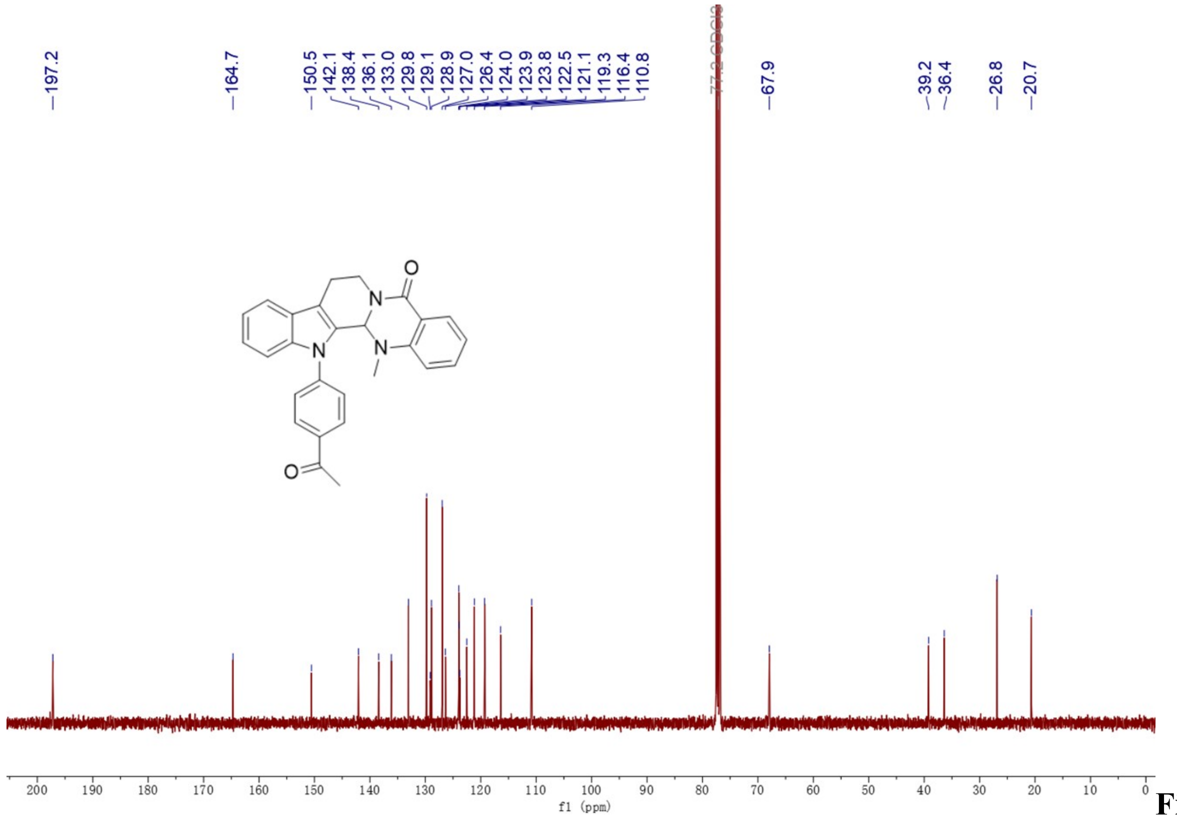

Figure 22. ^{13}C NMR spectrum of compound **3k** (100 MHz, $CDCl_3$)

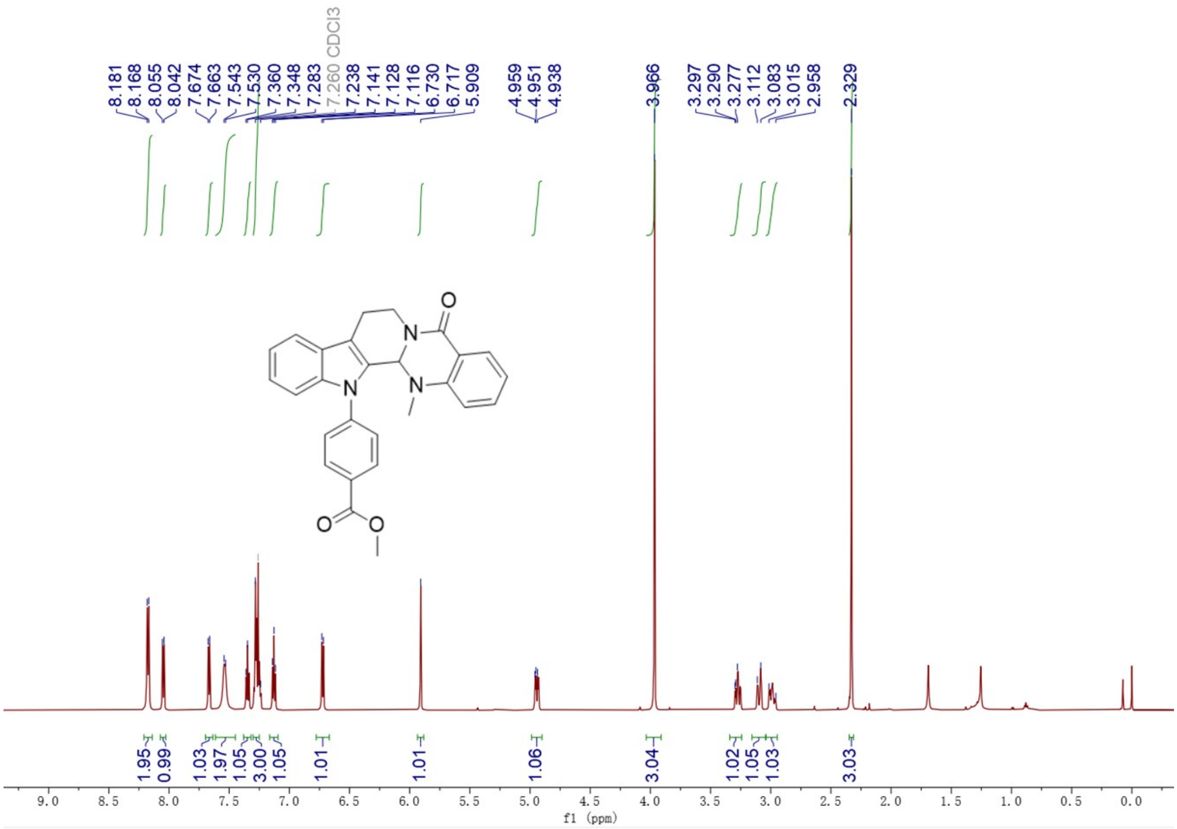

Figure 23. ^{1}H NMR spectrum of compound **3l** (600 MHz, $CDCl_3$)

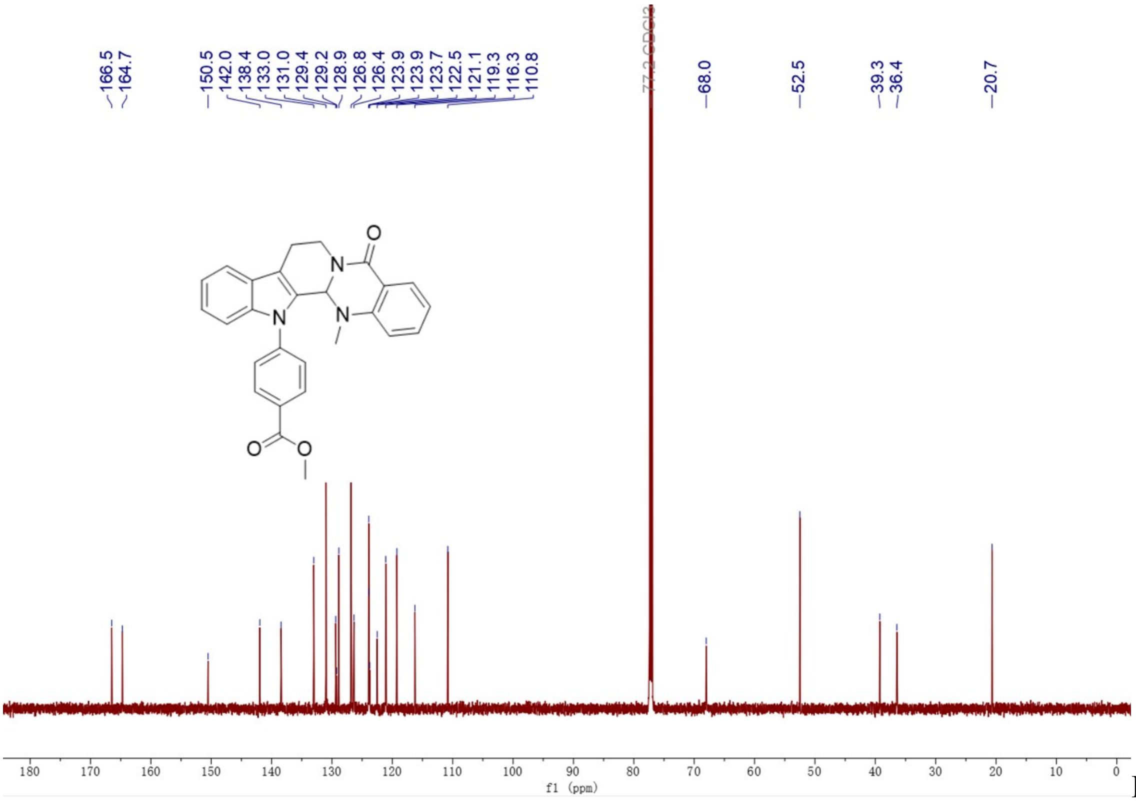

Figu

re 24. ^{13}C NMR spectrum of compound **3l** (150 MHz, $CDCl_3$)

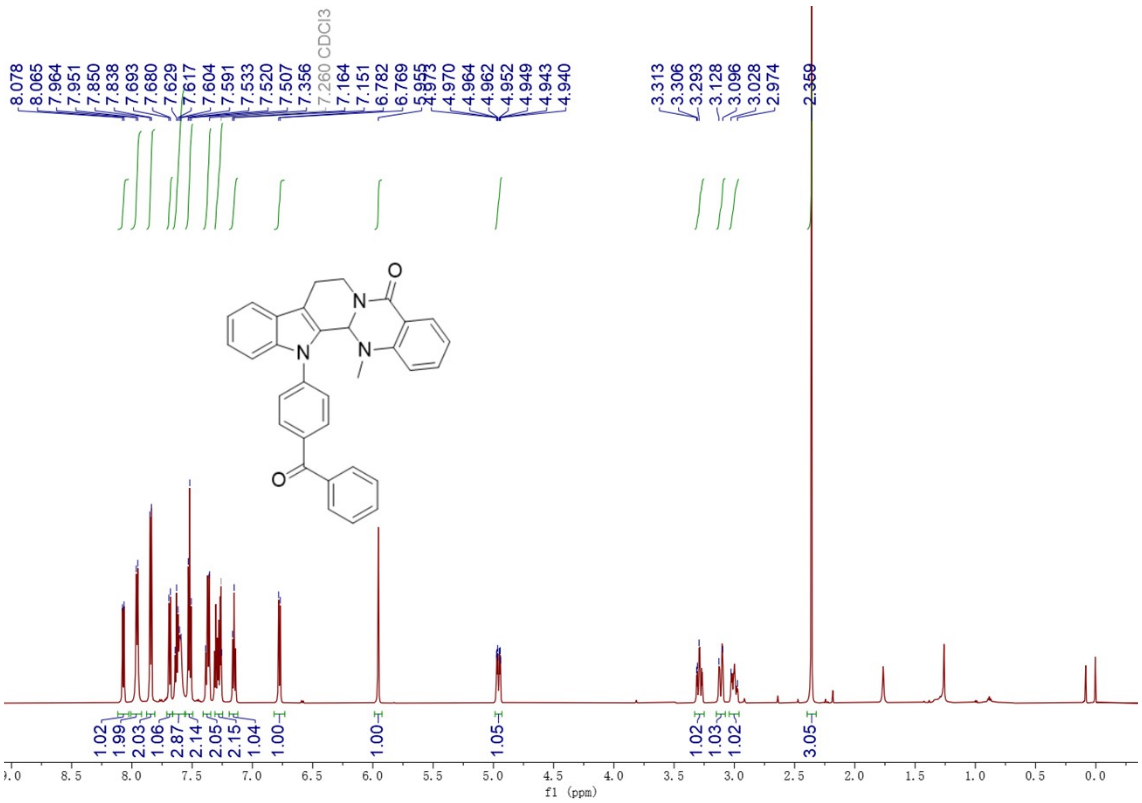

Figure 25. ^{1}H NMR spectrum of compound **3m** (600 MHz, $CDCl_3$)

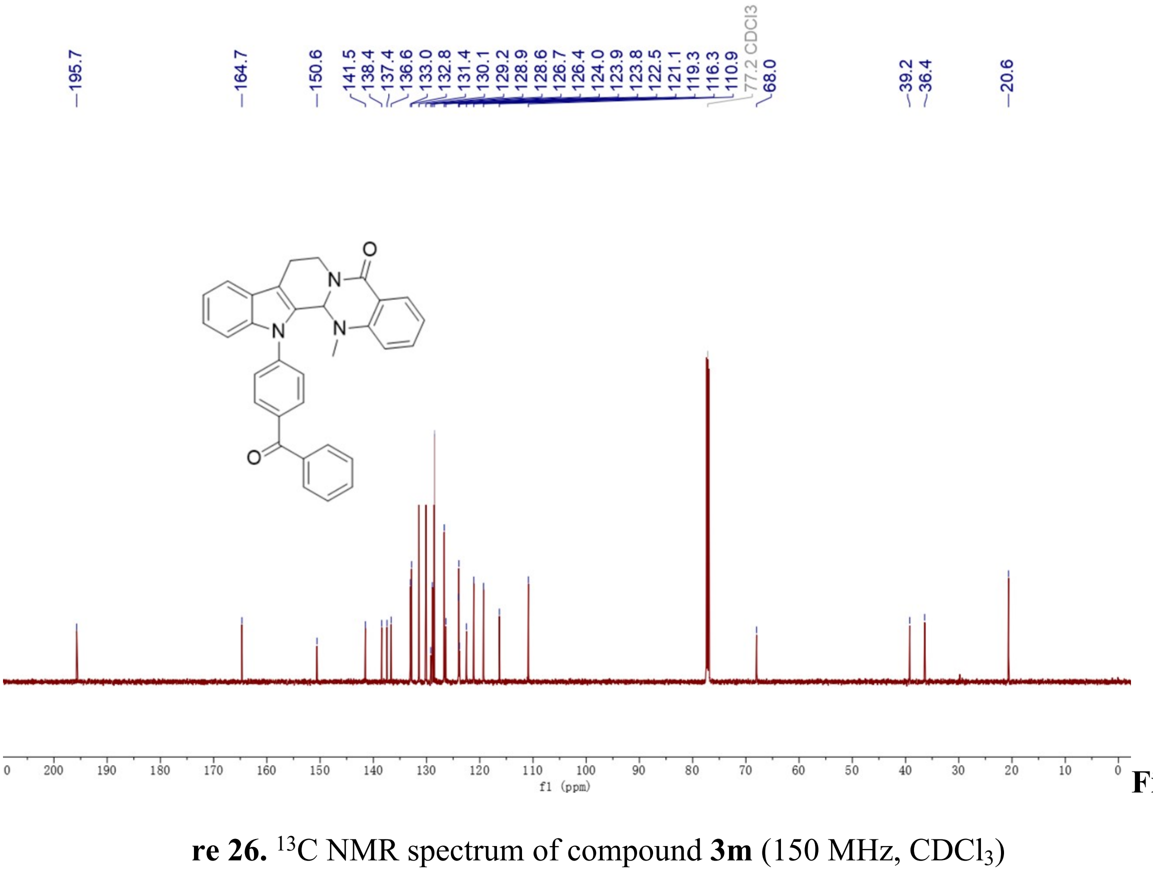

Figure 26. ^{13}C NMR spectrum of compound **3m** (150 MHz, $CDCl_3$)

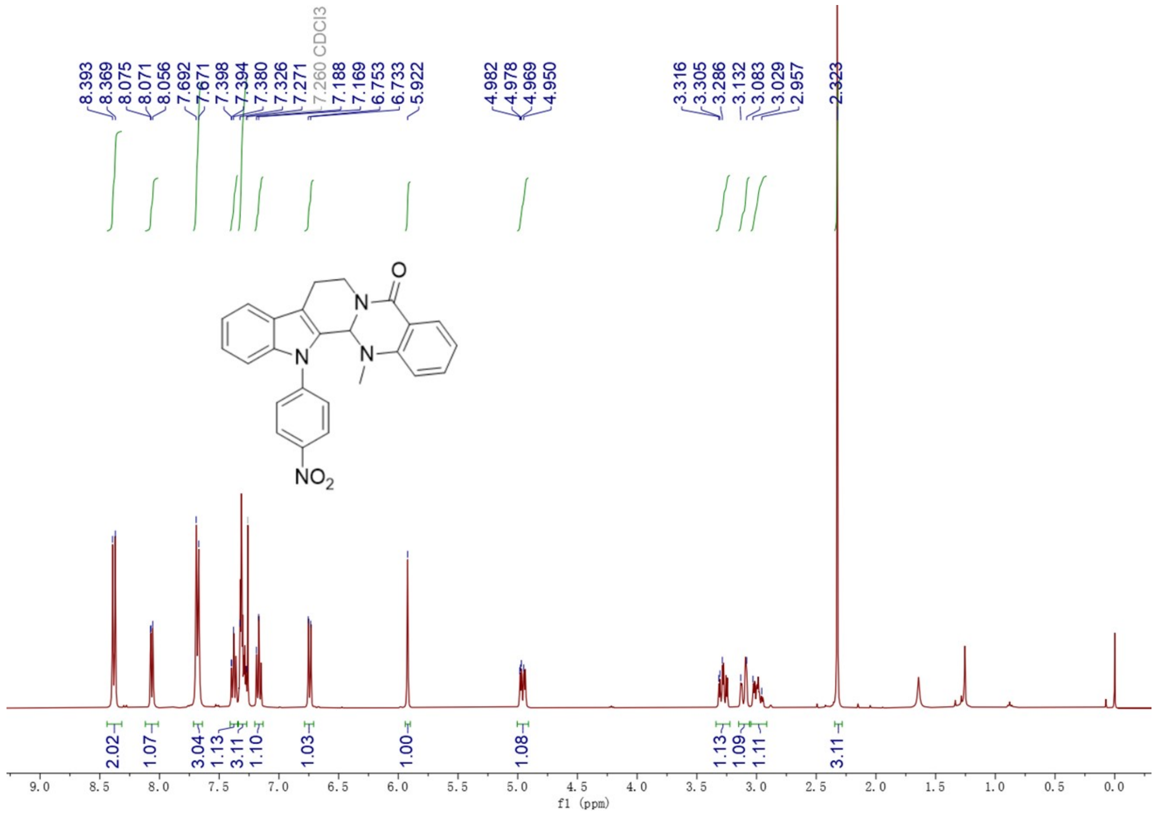

Figure 27. ^{1}H NMR spectrum of compound **3n** (400 MHz, $CDCl_3$)

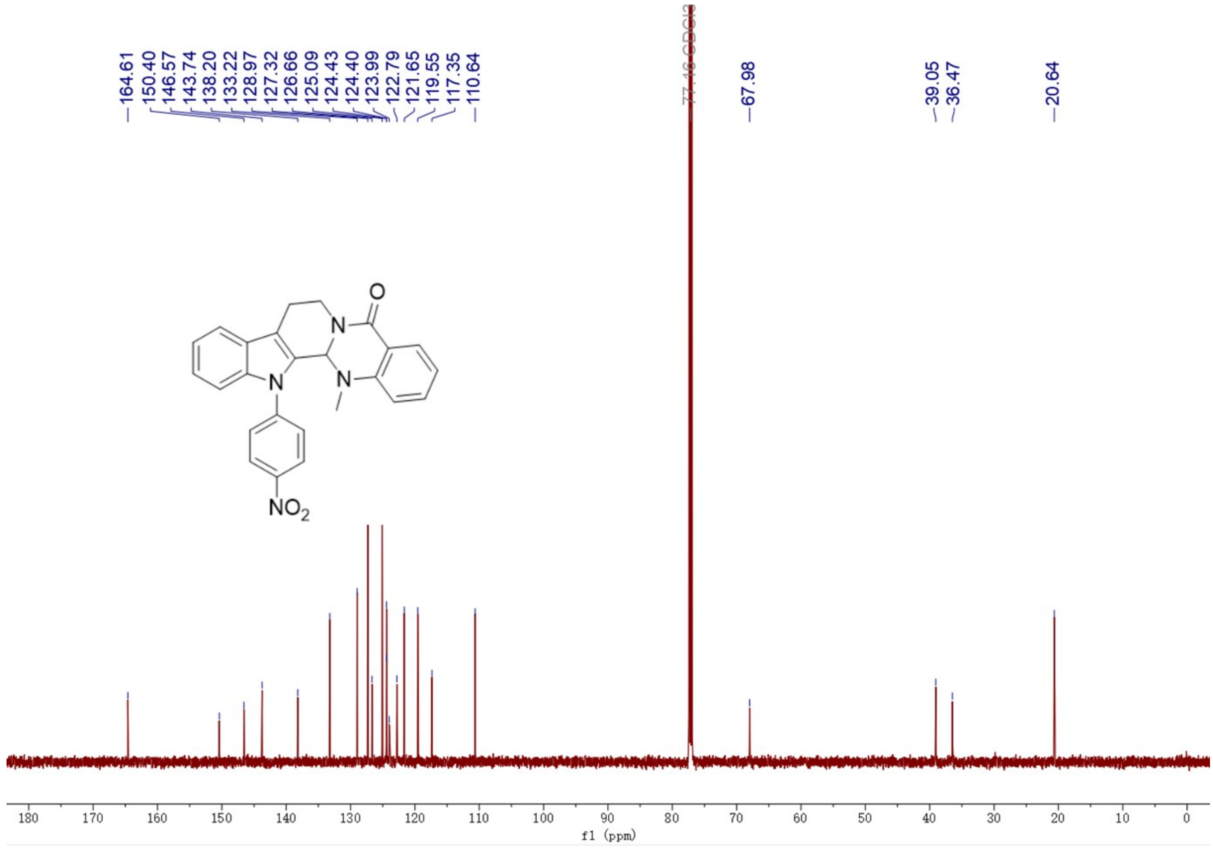

Figure 28. ^{13}C NMR spectrum of compound **3n** (150 MHz, $CDCl_3$)

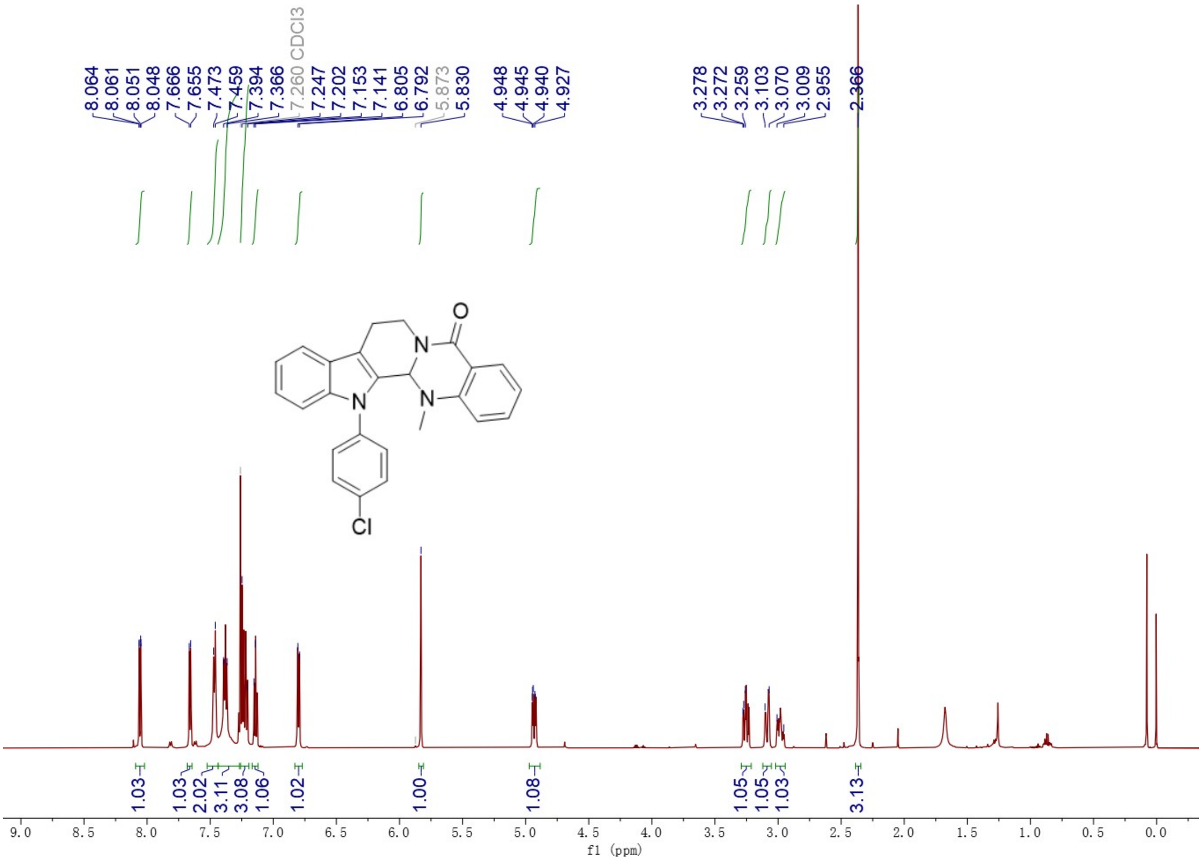

Figure 29. ^{1}H NMR spectrum of compound **3o** (600 MHz, $CDCl_3$)

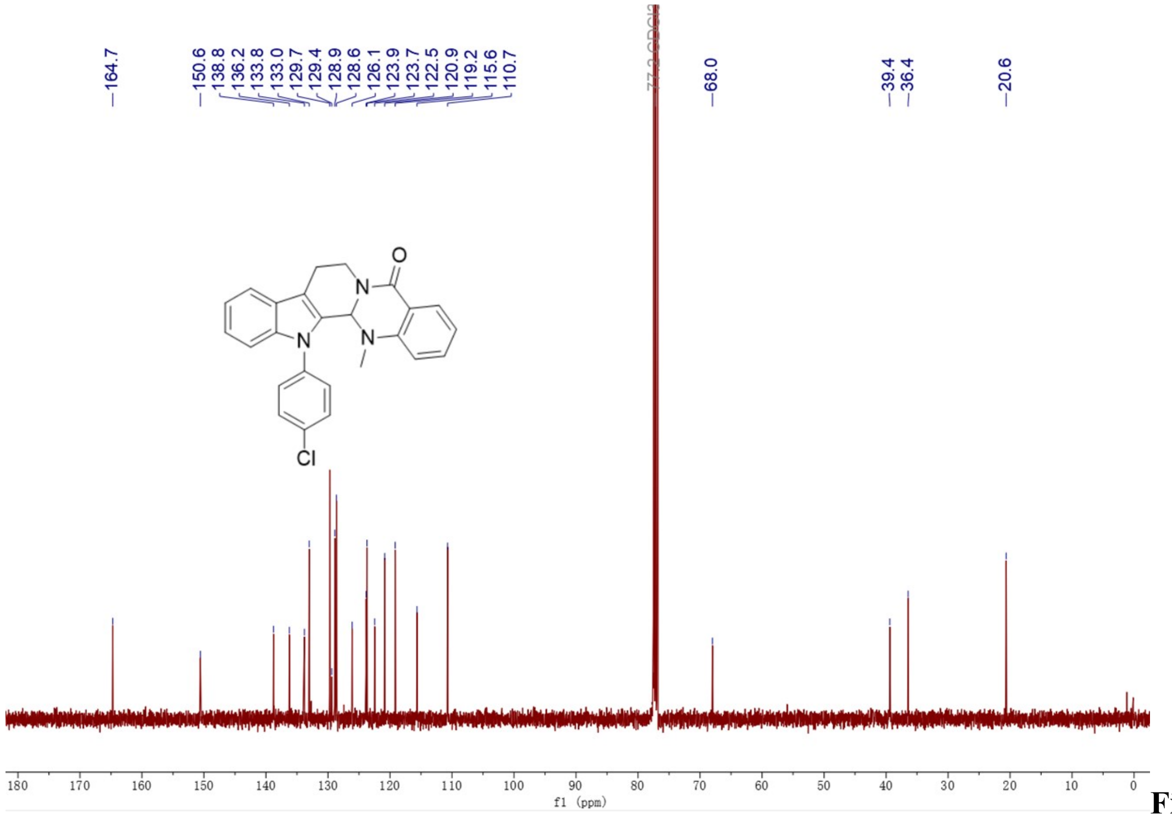

Fig

ure 30. ^{13}C NMR spectrum of compound **3o** (100 MHz, $CDCl_3$)

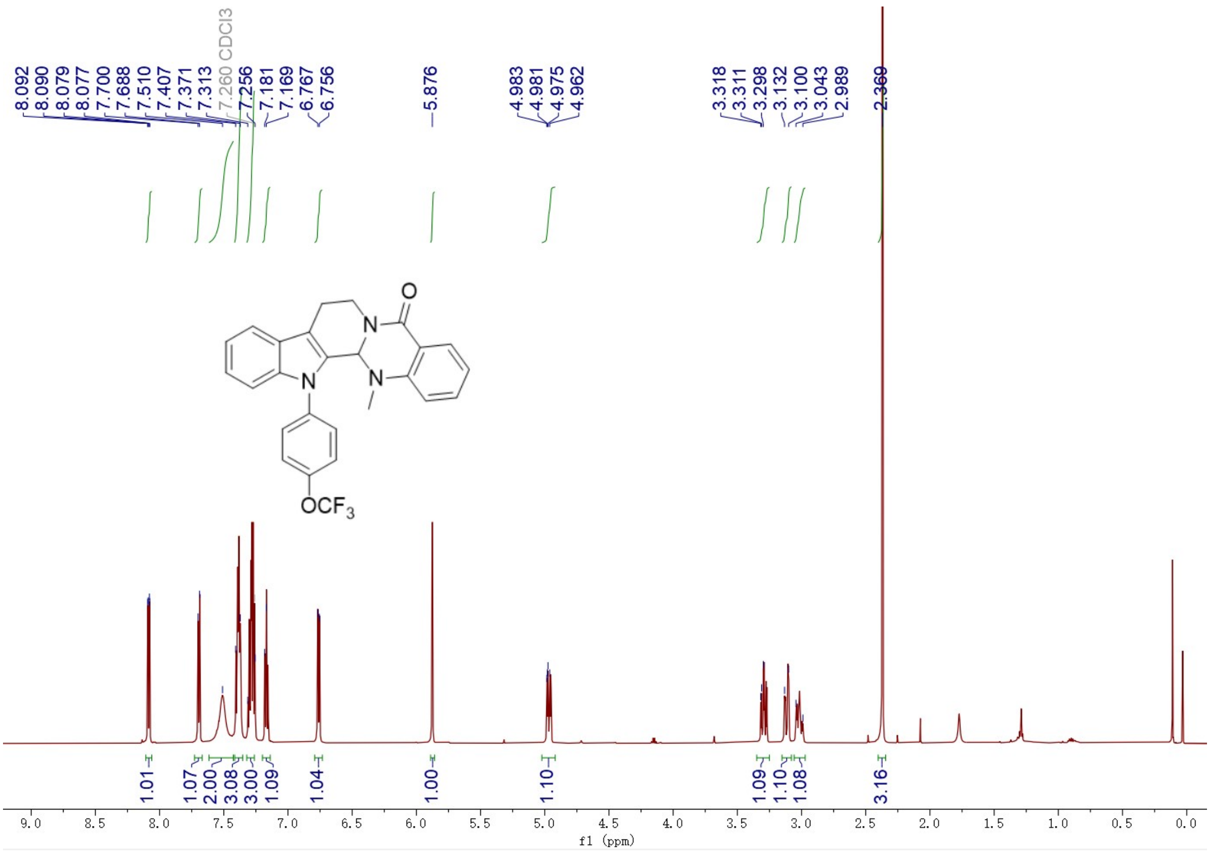

Figure 31. ^{1}H NMR spectrum of compound **3p** (600 MHz, $CDCl_3$)

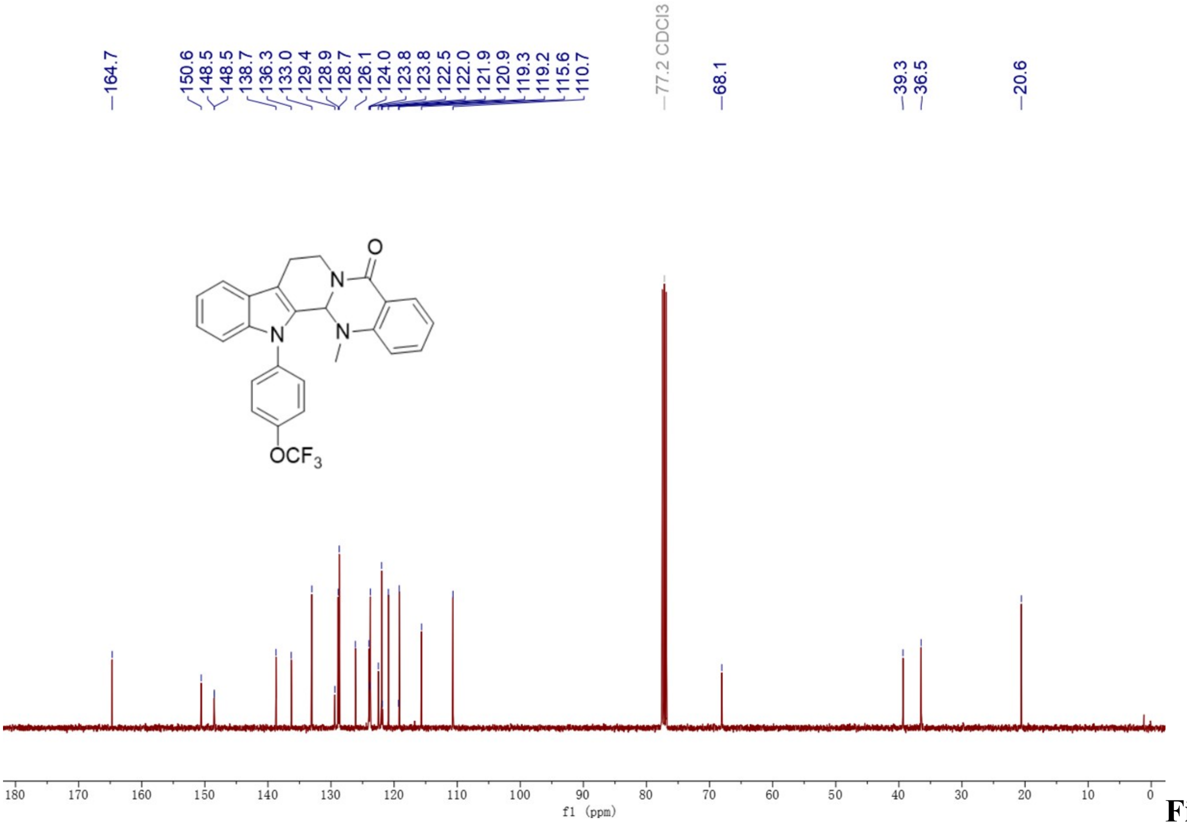

Figure 32. ^{13}C NMR spectrum of compound **3p** (100 MHz, $CDCl_3$)

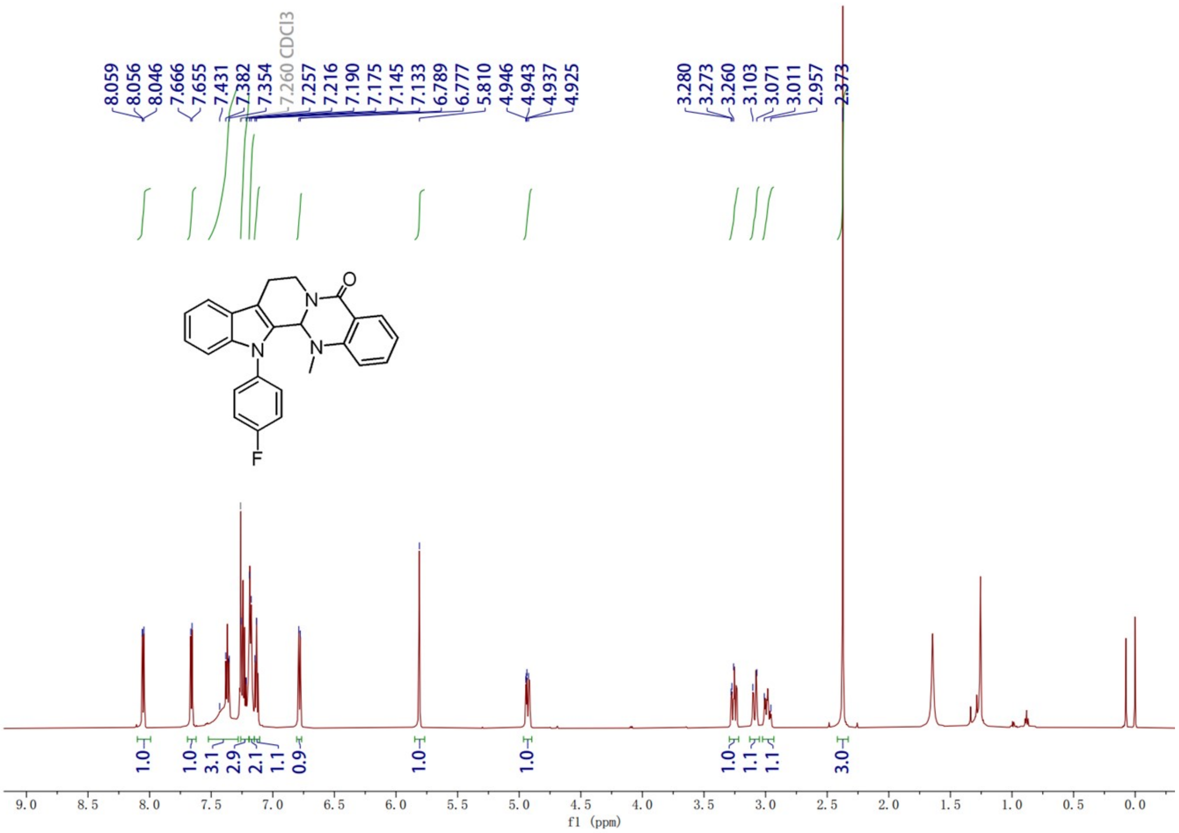

Figure 33. ^{1}H NMR spectrum of compound **3q** (600 MHz, $CDCl_3$)

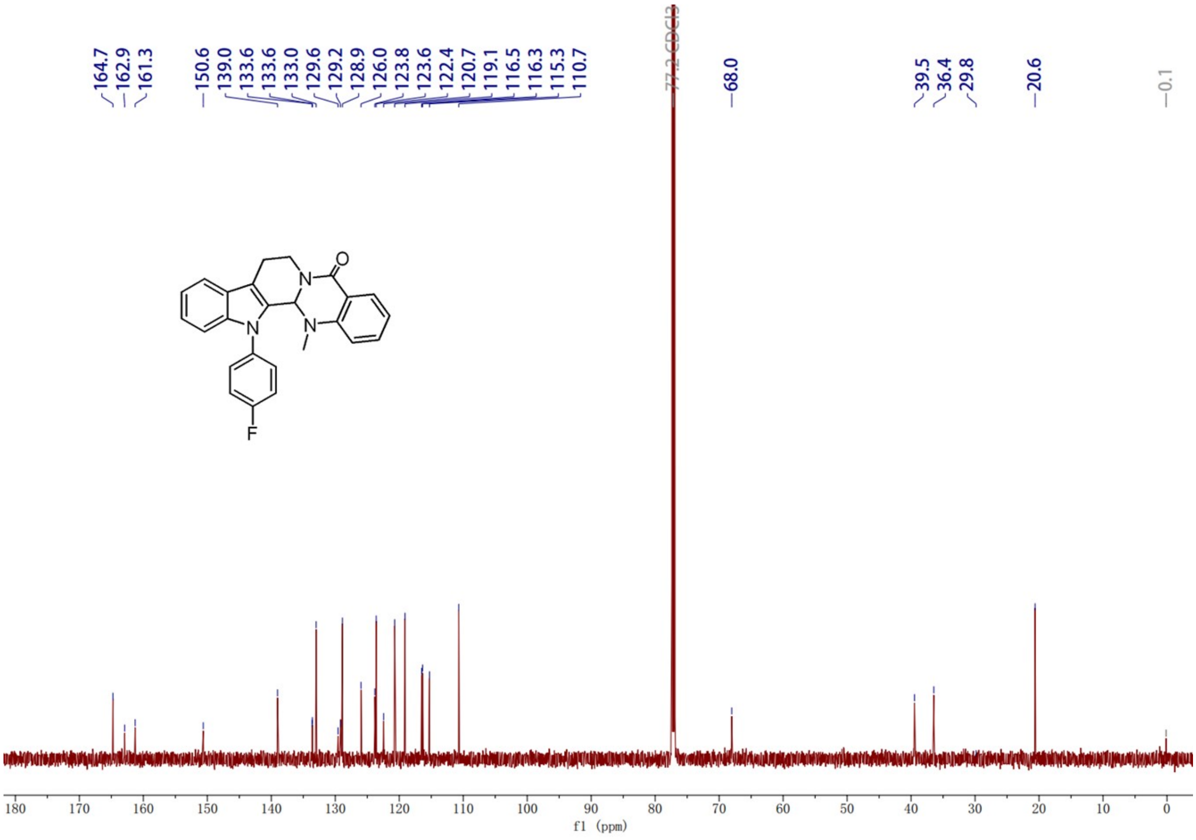

Figure 34. ^{13}C NMR spectrum of compound **3q** (150 MHz, $CDCl_3$)

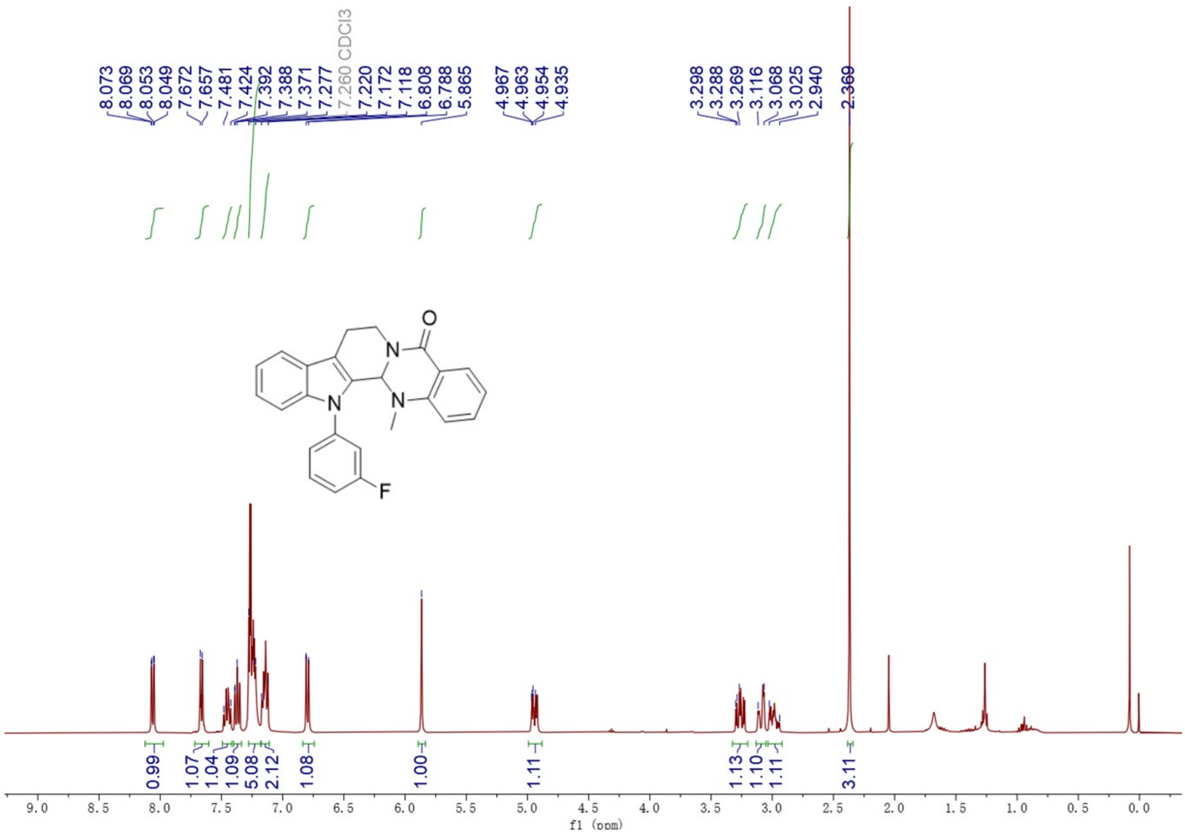

Figure 35. ^{1}H NMR spectrum of compound **3r** (400 MHz, $CDCl_3$)

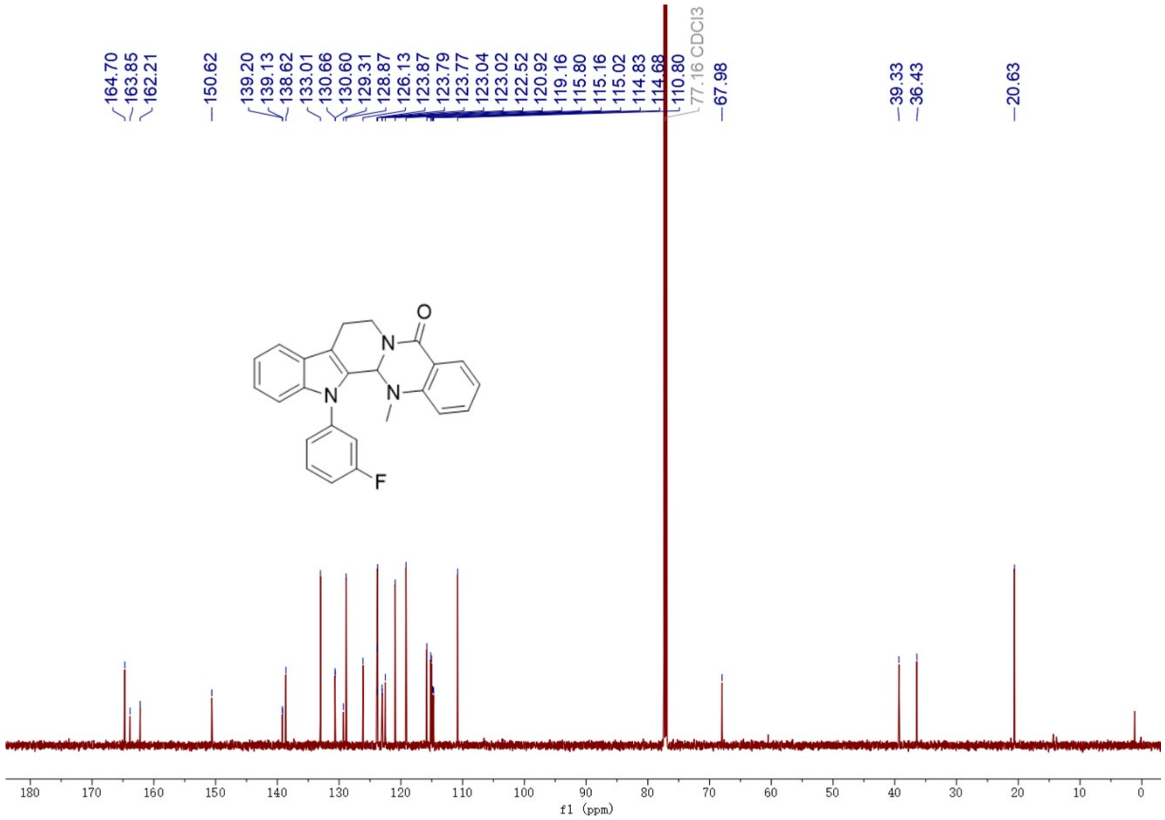

Figure 36. ^{13}C NMR spectrum of compound **3r** (150 MHz, $CDCl_3$)

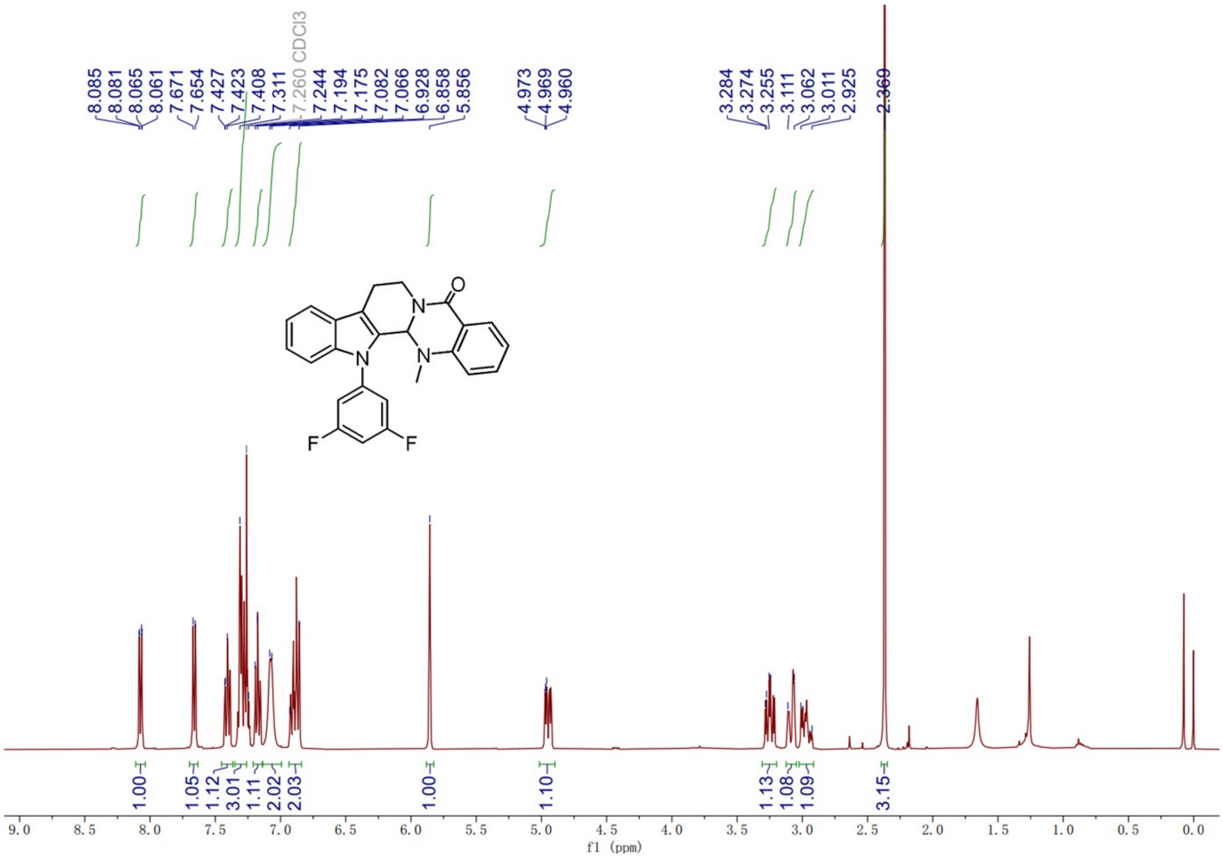

Figure 37. ^{1}H NMR spectrum of compound **3s** (400 MHz, $CDCl_3$)

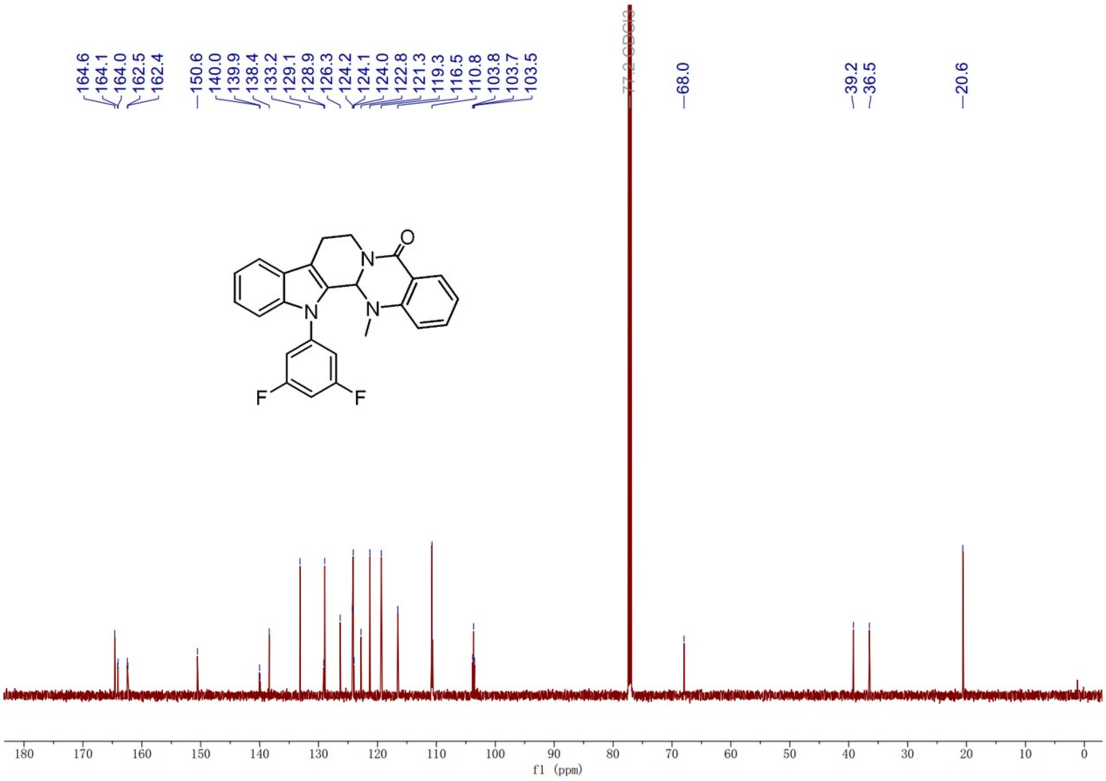

Figure 38. ^{13}C NMR spectrum of compound **3s** (150 MHz, $CDCl_3$)

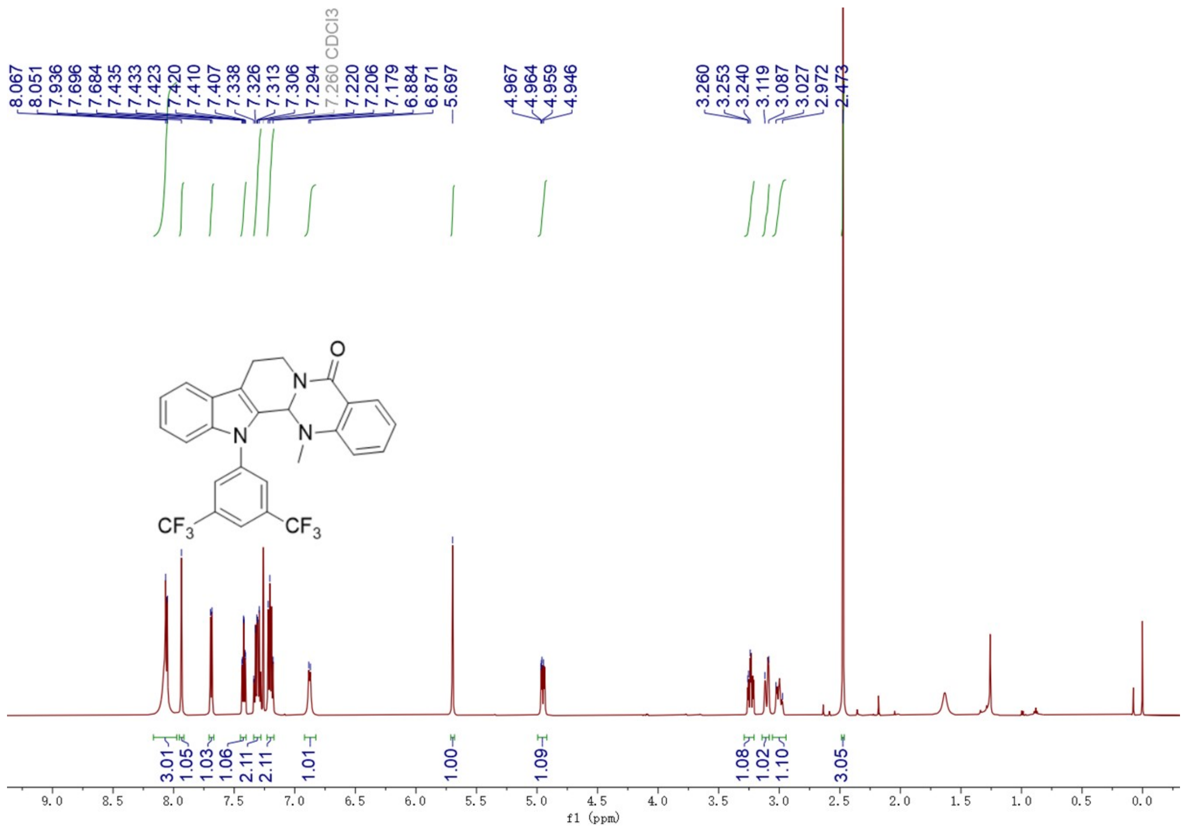

Figure 39. ^{1}H NMR spectrum of compound **3t** (600 MHz, $CDCl_3$)

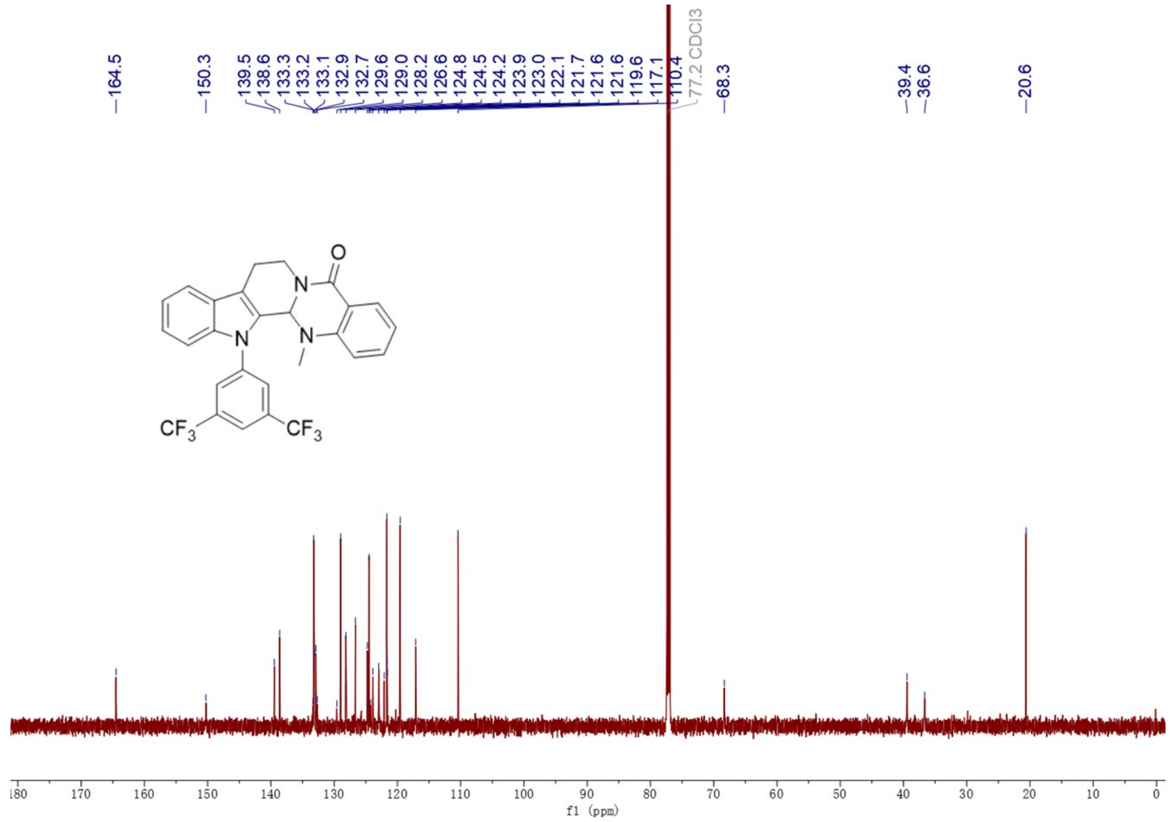

Figure 40. ^{13}C NMR spectrum of compound **3t** (150 MHz, $CDCl_3$)

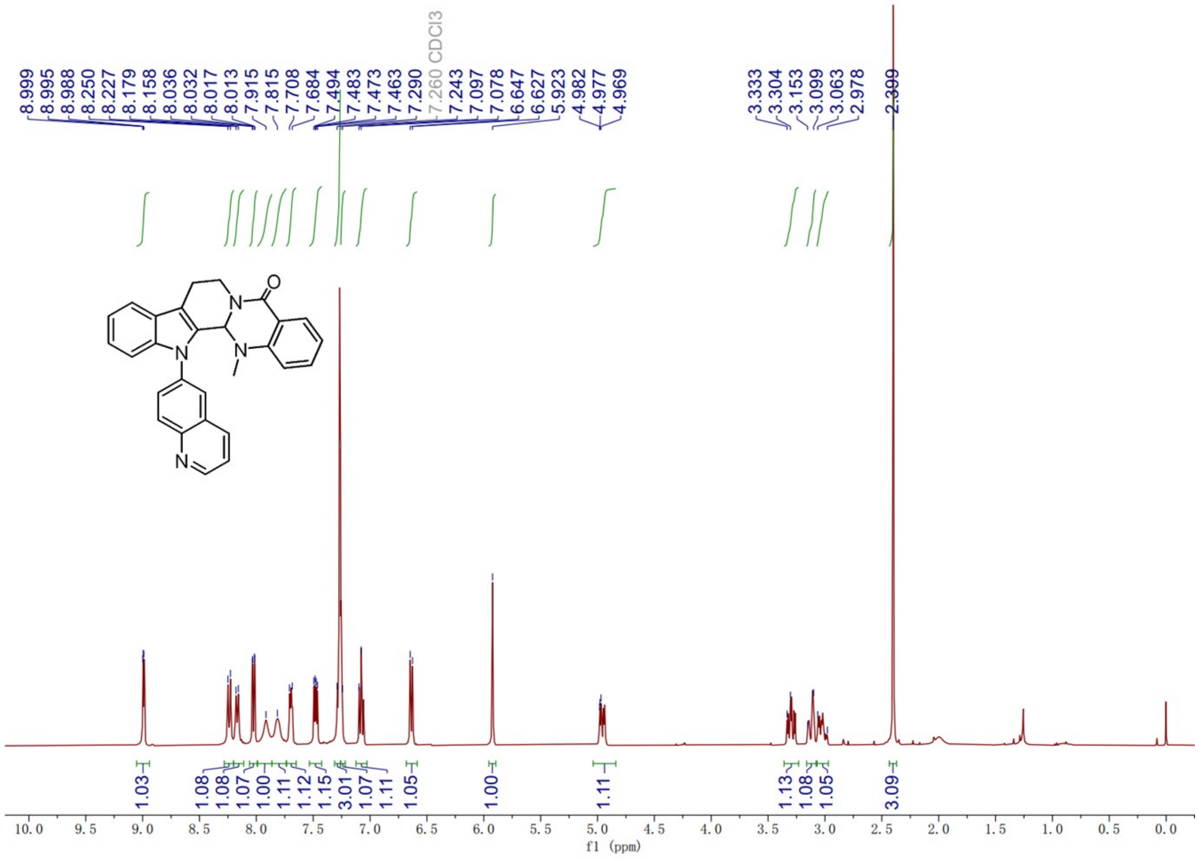

Figure 41. ^{1}H NMR spectrum of compound **3u** (400 MHz, $CDCl_3$)

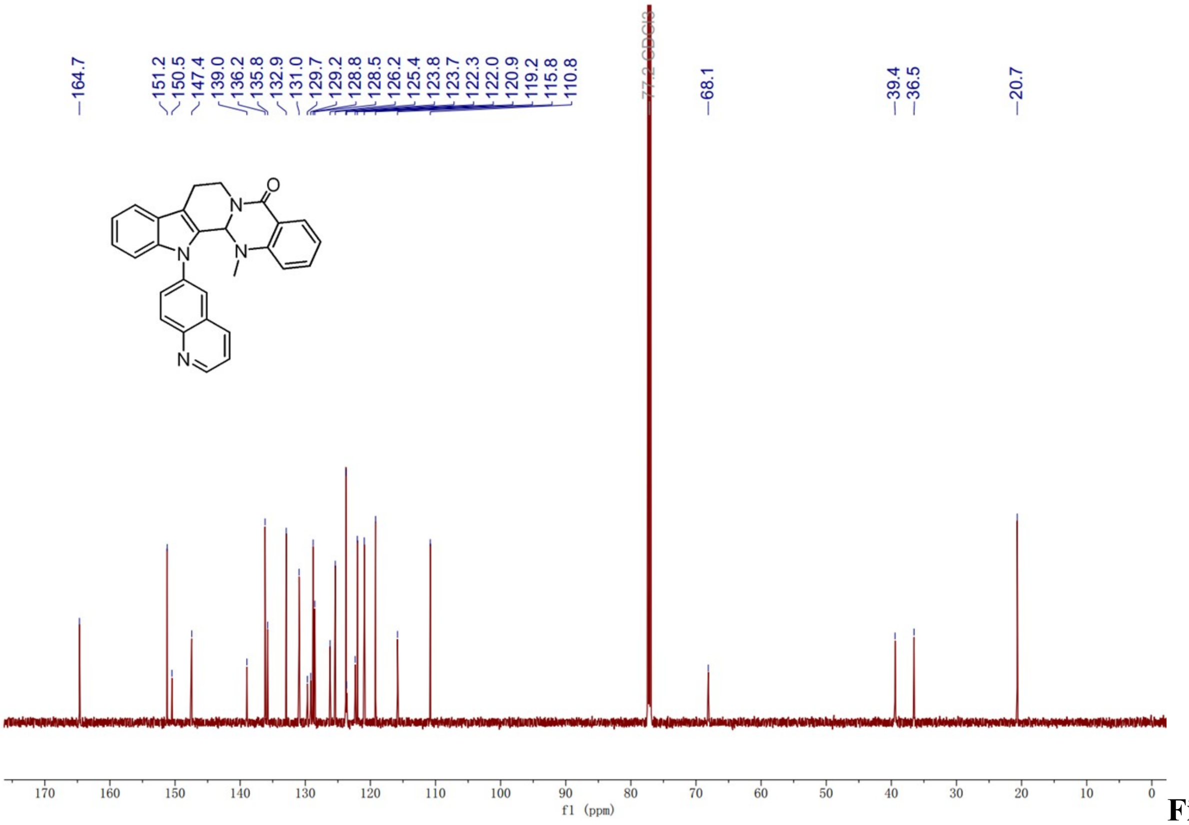

Fi

gure 42. ^{13}C NMR spectrum of compound **3u** (150 MHz, $CDCl_3$)

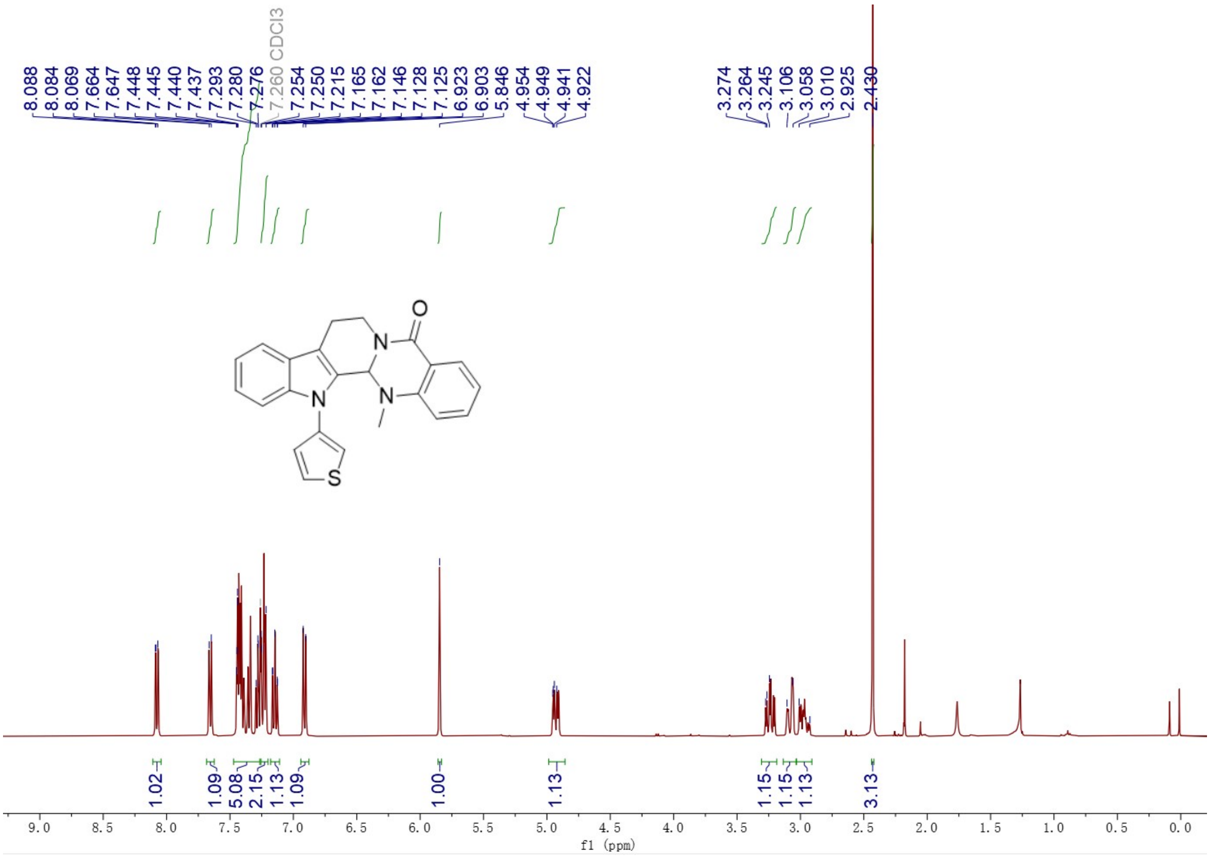

Figure 43. ^{1}H NMR spectrum of compound **3v** (400 MHz, $CDCl_3$)

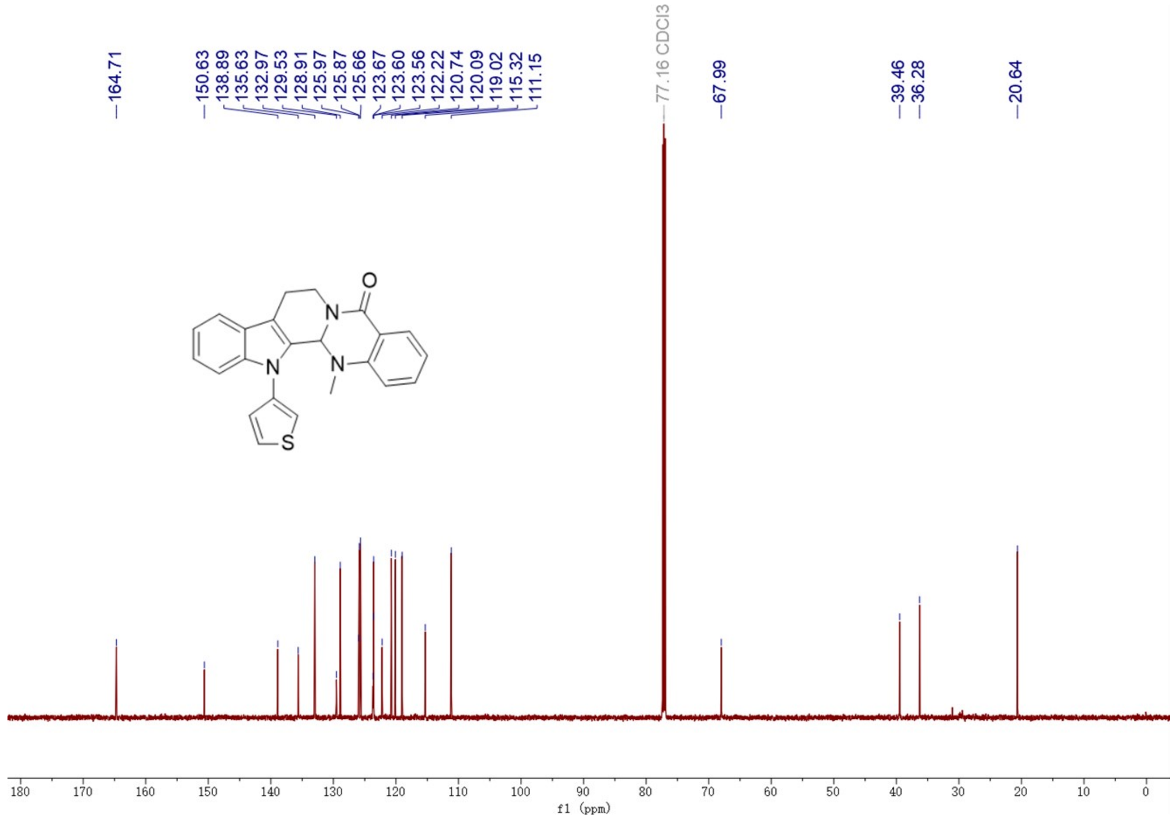

Figure 44. ^{13}C NMR spectrum of compound **3v** (150 MHz, $CDCl_3$)
